# Functional Group Transformation Approach to Chemically Recyclable Polymers from Ultra-Low to Moderate Strain Monomers

**DOI:** 10.1021/acs.macromol.4c03248

**Published:** 2025-04-11

**Authors:** Tarek Ibrahim, Kaia Kendzulak, Angelo Ritacco, Melanie Monetti, Hao Sun

**Affiliations:** Department of Chemistry and Chemical & Biomedical Engineering, Tagliatela College of Engineering, University of New Haven, West Haven, Connecticut 06516, United States

## Abstract

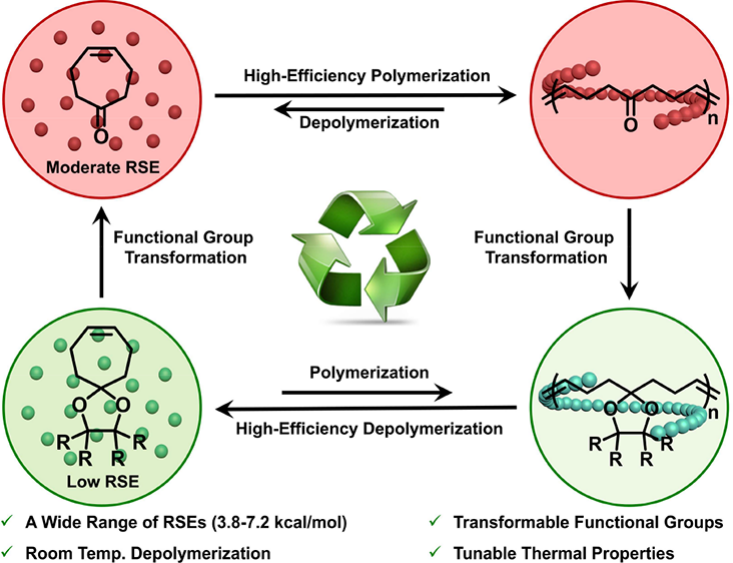

Ring-opening metathesis polymerization (ROMP) has been widely used for the synthesis of functional polymers. However, most ROMP-derived polymers are nondepolymerizable, limiting their sustainability and eco-friendiness. While recent advances in designing low-strain cyclic olefin monomers have enabled the ROMP synthesis of depolymerizable polyolefins, the scope of these monomers remains limited due to the narrow range of ring strain energies (RSEs = 4.7–5.4 kcal/mol) required to allow both polymerization and depolymerization in a closed-loop recycling process. Herein, we present a new class of chemically recyclable polyolefins based on cycloheptene derivatives with RSEs ranging from 3.8 to 7.2 kcal/mol. The wide range of RSEs enabled the establishment of a structure–polymerizability–depolymerizability relationship, shedding light on the role of RSE in both polymerization and depolymerization. A functional group transformation (FGT) strategy, harnessing reversible ketone-to-acetal chemistry, was developed to overcome the low polymerizability of low-strain monomers and the moderate depolymerizability of polymers made from moderate-strain monomers. This FGT approach not only enhanced the chemical recycling of moderately depolymerizable polyolefins but also provided access to highly depolymerizable polyolefins that are challenging to synthesize through direct ROMP of ultralow strain monomers. Moreover, the thermal properties of the chemically recyclable polyolefins developed in this study are highly tunable, with a broad range of glass transition temperatures (−7 to 104 °C), highlighting their potential for various applications.

## Introduction

Chemical recycling to monomers (CRM) represents an ideal approach to enhancing the sustainability and environmental friendliness of polymer materials.^1,2^ In a typical CRM process, postconsumer polymer waste is depolymerized into its constituent monomers, which can then be repolymerized into new polymer products with properties comparable to the original materials.^3−5^ Moreover, the CRM approach can effectively recycle mixed or contaminated plastics that can not be processed by traditional mechanical recycling method.^6^ Despite its significant promise, chemical recycling of commodity polymers, especially vinyl polymers with high ceiling temperatures, is quite energy-intensive and often requires high temperatures (>400 °C) to initiate the depolymerization process.^7^ Therefore, it would be highly desirable to develop new technologies to make chemical recycling process energetically efficient and economically viable.

Two approaches are currently being pursued to enhance the chemical recyclability of polymers. The first one involves facilitating the depolymerization of existing commodity polymers by designing novel catalytic systems, such as photocatalysts.^8^ Compared to traditional thermally induced depolymerization, the photoassisted process has demonstrated high efficiency in achieving relatively lower-temperature depolymerization of various commodity vinyl polymers, including polymethacrylates and polystyrene.^9−12^ However, it should be noted that high reaction temperatures (>100 °C) are still required when light is used to assist the solution depolymerization of these vinyl polymers, due to their inherently high ceiling temperatures.^9^ The second approach focuses on the design of novel polymer structures that can be easily depolymerized. Over the past decade, significant progress has been made in developing new depolymerizable polymer structures, driven by various chain-growth polymerization techniques, such as radical polymerization,^13−18^ ionic ring-opening polymerization,^2,19−24^ nucleophilic aromatic ring-opening polymerization,^25^ coordination ring-opening polymerization,^26−28^ and ring-opening metathesis polymerization (ROMP).^29−40^ Among these depolymerizable polymers, polyolefins produced via ROMP have attracted increasing interest due to their hydrolytically stable backbones and mild depolymerization conditions.^41^

**Figure 1:**
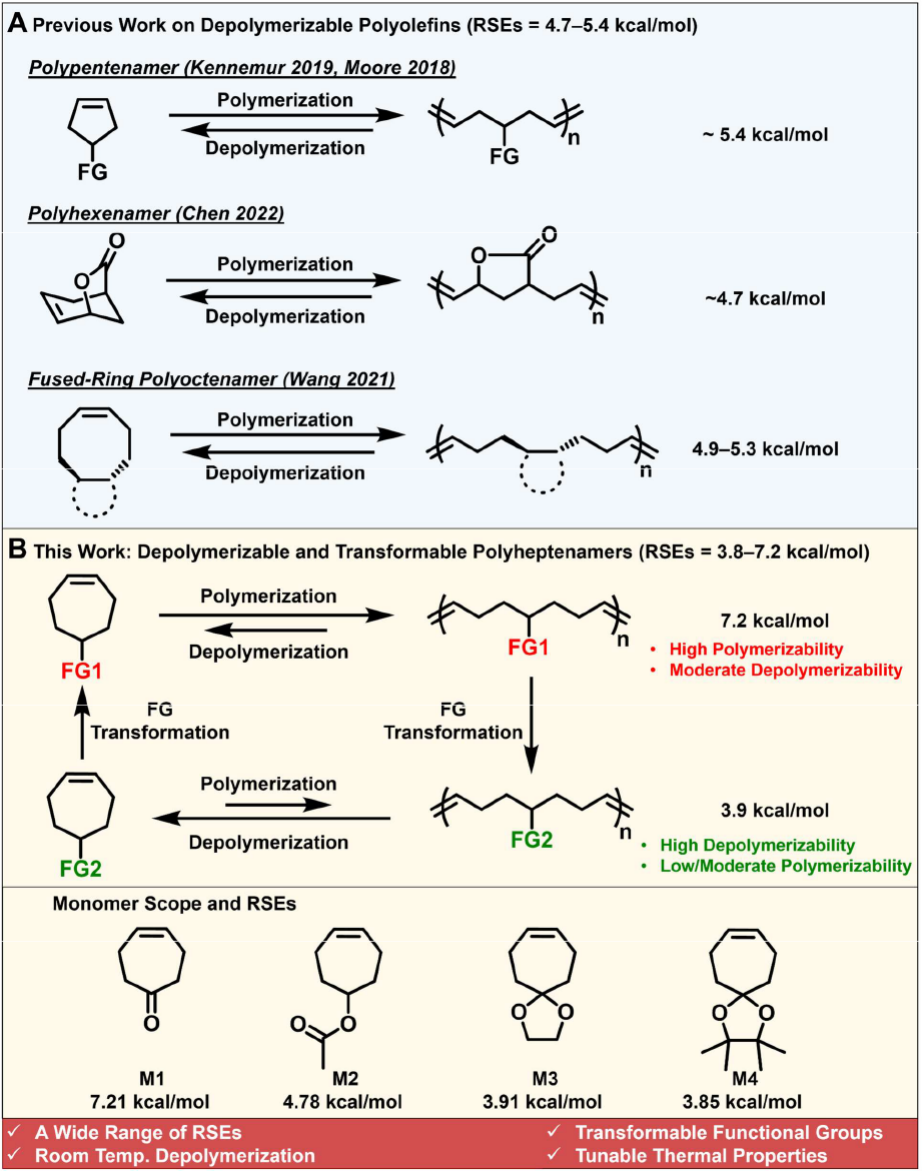
Depolymerizable polyolefins and the ring strain energies of their constituent monomers. (A) Representative examples of depolymerizable polyolefins based on various low-strain cyclic olefin monomers. (B) This study: depolymerizable and transformable polyheptenamers synthesized from ultralow to moderate strain monomers.

Cyclic olefin monomers with low ring strain energies (RSEs) have recently been employed in ROMP to produce depolymerizable polyolefins (Figure 1A).^42^ Examples include cyclopentene derivatives,^29,30,43^ 2,3-dihydrofuran,^36^ cyclohexene derivatives,^37−39^ cycloheptene,^44^ and fused-ring cyclooctenes.^31,33−35,45^ The low strain of these monomers facilitates the efficient depolymerization of polyolefins through a ring-closing metathesis process. Nevertheless, the scope of monomers for producing chemically recyclable polyolefins remains limited, since a suitable range of ring strain (4.7–5.4 kcal/mol) is essential for achieving both polymerization and depolymerization in a closed-loop recycling process.^41,42^ Indeed, polymerization has proven rather challenging for ultralow-strain monomers such as cyclohexene (RSE = 2.5 kcal/mol),^38,46^ while depolymerization is hindered by the moderate RSEs of monomers such as cyclooctene (RSE = 8.2 kcal/mol).^33^ Therefore, previous studies have focused on designing or discovering cyclic olefin monomers that fall within the narrow RSE window required for producing depolymerizable polymers.^33,39^

In this study, we designed and synthesized a series of functional cycloheptene-derived monomers (M1–M4) with a wide range of RSEs from 3.85 to 7.21 kcal/mol (Figure 1B). Solvent-free ROMP of these cycloheptene-derived monomers led to functional polymers that are depolymerizable under mild conditions. It was observed that RSE plays a pivotal role in governing the polymerizability of monomers and the depolymerizability of their corresponding polymers. To address the low polymerizability of low-strain monomers (M3 and M4) and the moderate depolymerizability of polyolefins produced from moderate-strain monomer (M1), we developed a functional group transformation (FGT) strategy to enable the reversible transformations of these monomers and their polymer structures (Figure 1B). This approach not only enhances the chemical recycling of moderately depolymerizable polymers by converting them into highly depolymerizable structures, but also facilitates the synthesis of highly depolymerizable polyolefins that are otherwise difficult to produce directly from ROMP of ultralow strain monomers. Moreover, these chemically recyclable polyolefins exhibit tunable thermal properties, with glass transition temperatures ranging from −7 to 104 °C, highlighting their potential for diverse industrial applications.

## Results and Discussion

### Design and Computational Analysis of Monomers

We began our preliminary exploration with the design of functional cycloheptene monomers. A library of monomers bearing ketone (M1), ester (M2), and acetal groups (M3 and M4) was designed and analyzed using density functional theory (DFT) to estimate their ring strain energies (Figures 1B, S1–S4, and Supporting Sections 3.1 and 5). DFT calculations revealed a significant impact of functional group on the ring strain. As the size of functional group increases, the RSE of monomer gradually decreases (Figure 1B). Specifically, M1, with a small ketone group, has a moderate RSE of 7.21 kcal/mol, whereas M4, which contains a bulky acetal group, exhibits an ultralow RSE of 3.85 kcal/mol.

**Figure 2:**
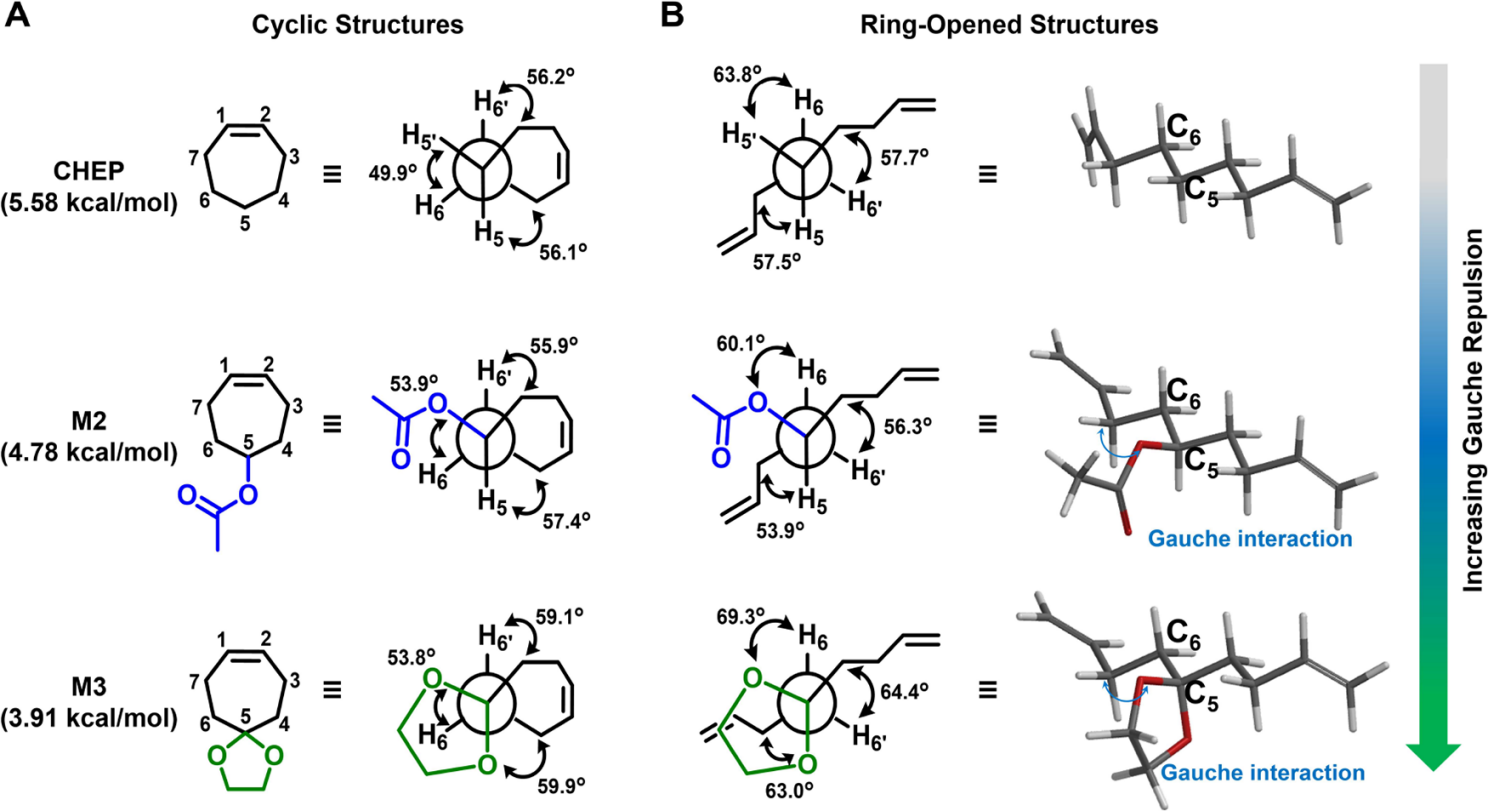
Newman projections of cycloheptene-derived monomers and their ring-opened structures. (A) The structures and their corresponding Newman projections for cycloheptene (CHEP) and functional monomers (M2 and M3). (B) Newman projections and their simulated ring-opened structures. Geometry optimizations were performed using the B3LYP/6-31G* level of theory in vacuum. The lowest-energy conformers were used for the Newman projection analysis.

To uncover the origin of the substituent effect on RSE, we examined the structures of monomers and their ring-opened forms via the Newman projections along the C5–C6 bond (Figures 2 and S5). Cycloheptene (CHEP) was used as a reference for comparison with the functional monomers. As shown in Figure 2A, the dihedral angles H5′–C5–C6–H6, C4–C5–C6–H6′, and H5–C5–C6–C7 in CHEP closely align with their corresponding dihedral angles (i.e., O–C5–C6–H6, C4–C5–C6–H6′, and H5–C5–C6–C7) in M2, indicating that substituting H5′ with an acetate group does not alter the ring structure. Similarly, no noticeable structural change in the ring was observed in M3, where both H5 and H5′ are replaced by a cyclic acetal group. We then analyzed the ring-opened structures of these monomers (Figure 2B). The dihedral angles remain similar for all the acyclic structures, regardless of the substitutions on C5. Notably, the ring-opening of functional monomers (M2 and M3) results in a significant gauche interaction between the substituent and an allyl group, destabilizing the structure due to the steric repulsion between these two bulky groups (Figure 2B). Since RSE represents the relative energy difference between the cyclic monomer and their ring-opened form (RSE = *H*_monomer_ – *H*_ring-opened form_ – *H*_ethylene_), an increase in the energy of the ring-opened form would result in a reduction of the RSE. As the size of the functional group increases, the gauche interaction becomes more significant, thereby leading to a smaller RSE.

### Synthesis of Monomers and Polymers

**Figure 3:**
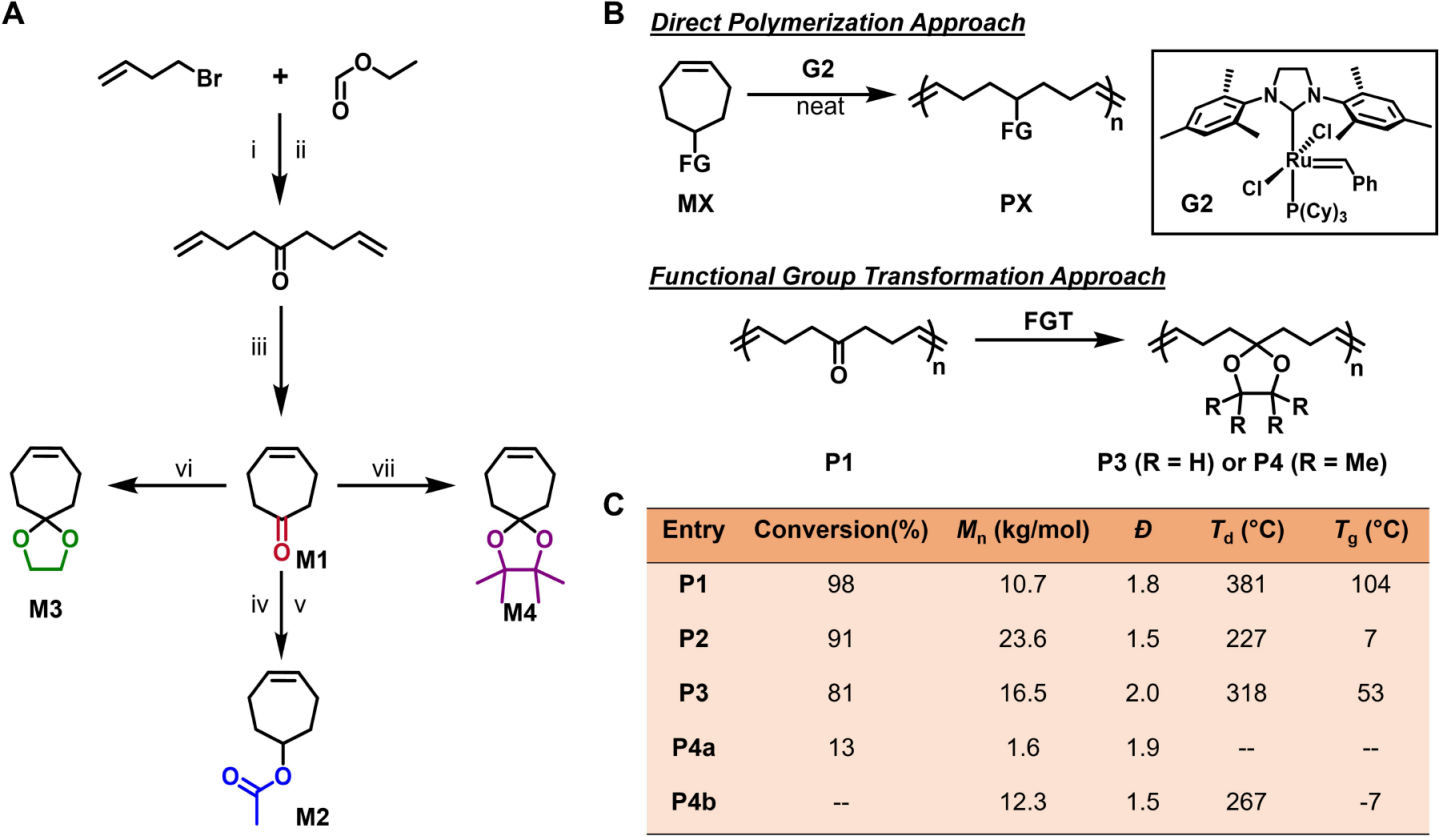
Synthesis and characterization of cycloheptene-based monomers and polymers. (A) Synthetic routes to monomers. M1 can be synthesized under three-step conditions: (i) Mg, THF, 0 °C; (ii) Jones’ reagent, acetone, 20 °C; and (iii) G2, DCM (20 mM), 40 °C. M1 can be subsequently converted into M2–M4 following these conditions (iv) NaBH_4_, THF/MeOH, 0–20 °C; (v) Ac_2_O, TEA, DMAP, DCM, 0–20 °C; (vi) ethylene glycol, *p*-TsOH, triethyl orthoformate, DCM, 40 °C; and (vii) pinacol, *p*-TsOH, triethyl orthoformate, DCM, 40 °C. (B) Two synthetic approaches to polymers: direct ring-opening metathesis polymerization (ROMP) and postpolymerization modification. (C) Polymer information. P1, P2, P3, and P4a were synthesized by direct ROMP of monomers (see Table S1). P4b was obtained via functional group transformation (FGT) of P1.

Since the ketone monomer (M1) serves as a precursor for the synthesis of other monomers, we first prepared M1 using a method described in a previous study (Figure 3A).^47^ M2 was subsequently obtained through the hydride reduction of M1, followed by acetylation of the resulting hydroxyl group. The acetal monomers M3 and M4 were generated by reacting M1 with ethylene glycol and pinacol, respectively.

The structures of all monomers were confirmed by nuclear magnetic resonance (NMR) spectroscopy and mass spectrometry (Figures S6–S19).

ROMP of monomers was enabled by Grubbs’ second-generation catalyst (G2). To optimize the polymerization conditions, we investigated the effects of reaction temperature (20–40 °C) and monomer concentration on the ROMP of M1 (entries 1–4 in Table S1). As shown in Table S1, the conversion of M1 gradually increased as the reaction temperature decreased, suggesting that the polymerization is driven by enthalpy. Moreover, bulk polymerization at room temperature (20 °C) led to a near-quantitative conversion of M1. Based on these results, solvent-free and room temperature conditions were employed for the polymerization of other monomers (M2–M4) to maximize their conversions.

Because ring strain energy is the driving force for ROMP, we reasoned that a decrease in RSE would result in a reduced polymerizability of the monomers. Indeed, monomer conversions markedly decreased from 98% for M1 to 13% for M4 under the same polymerization conditions (i.e., solvent-free and room-temperature), confirming the important role of RSE in their polymerizability (entries 4–7 in Table S1). The resulting polymers (P1–P4a) were characterized by NMR (Figures S20–S25) and size exclusion chromatography (SEC) (Figures S27–S30). According to the SEC results, the molecular weight distributions of these polymers are relatively broad (*Đ* = 1.5–2.0), which stem from secondary metathesis events typically associated with the ROMP of low-to-moderate strain monomers.^48^

To overcome the low polymerizability of M4 (RSE = 3.85 kcal/mol), we further employed a functional group transformation approach based on ketone-to-acetal chemistry that efficiently converted P1 into P4b (Figure 3B). NMR analysis of P4b confirmed a quantitative transformation of ketone groups into acetals, demonstrating the robustness of this approach (Figure S26). Furthermore, the molecular weight of P4b is markedly higher than that of P4a, which was synthesized via the direct ROMP approach (Figures 3C, S30, and S31).

### Depolymerization Study of Polymers

Given that the polymer library (P1–P4) is derived from monomers with a broad range of RSEs (3.85–7.21 kcal/mol), we hypothesized that a structure-depolymerization relationship can be established by investigating their depolymerization behaviors. Depolymerization study was carried out under standard ring-closing metathesis conditions (20 mM olefin concentration with G2 as the catalyst). A kinetic study on the depolymerization of P3 revealed that an equilibrium, with over 96% of the monomers regenerated, was reached within 10 h (Figures S32).

**Figure 4:**
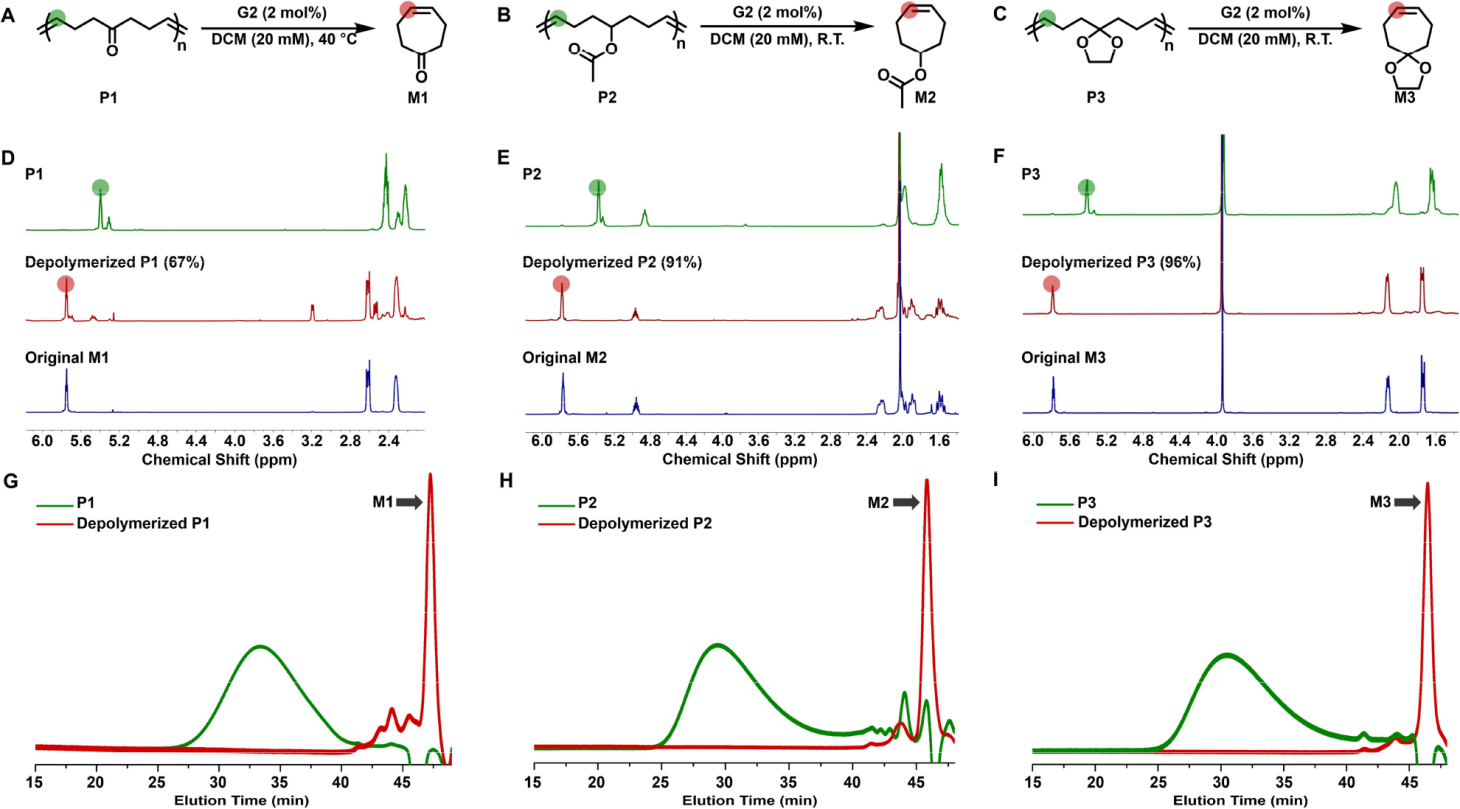
Depolymerization study of functional polyheptenamers. (A–C) Schematic illustrations of the depolymerization of P1–P3. Depolymerization experiments were conducted under dilute conditions (20 mM olefin) in the presence of G2 for 12 h. Depolymerization of P1 was performed at 40 °C, while depolymerizations of P2 and P3 were carried out at room temperature. (D–F) ^1^H NMR spectra of polymers (top green), depolymerized products (middle red), and original monomers (bottom blue). Monomer recovery yields were calculated based on NMR analysis. (G–I) Size exclusion chromatography traces of the polymers (green) and their depolymerized products (red).

To elucidate the impact of RSE on depolymerization efficiency, we examined the depolymerization performance of P1, P2, P3, and P4b (Figures 4 and S33–S36). As shown in Figure S33, depolymerization of P1 at room temperature resulted in only 44% monomer recovery. Increasing the reaction temperature to 40 °C enhanced the yield of monomer regeneration to 67%, while a noticeable amount of the thermally rearranged product, 3-cycloheptenone,^49^ was also formed (Figures 4A,D,G and S34). The moderate depolymerizability of P1 can be attributed to the moderate ring strain of M1 (7.21 kcal/mol). In comparison to P1, the depolymerization of polymers P2–P4 with lower RSEs (3.85–4.78 kcal/mol) gave rise to significantly higher monomer recovery yields (>90%), even under room-temperature conditions (Figures 4 and S37). NMR analysis revealed that the ^1^H NMR spectra of the depolymerization products of P2–P4 are nearly identical to those of their original monomers (Figures 4E,F and S36). In addition, SEC data corroborated the NMR results by showing the complete disappearance of polymer signals and the appearance of monomer peaks upon depolymerization (Figures 4H,I and S35). Critically, a reduction in RSE led to enhanced depolymerization efficiency, with P3 and P4 achieving monomer recovery yields exceeding 96% (Figure S37). These results unequivocally verify the role of low RSE in promoting ring-closing metathesis depolymerization.

**Figure 5:**
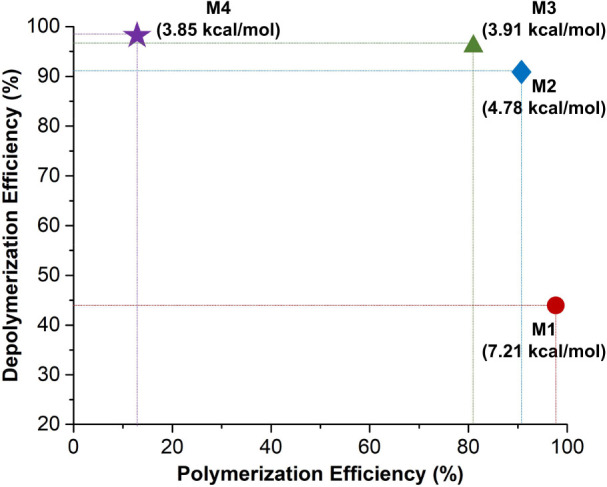
Structure–polymerizability–depolymerizability relationship of M1–M4 and their corresponding polymers P1–P4. Polymerization efficiency is defined as the monomer conversion achieved during bulk polymerization at 20 °C. Depolymerization efficiency is expressed as the yield of monomer regeneration obtained during depolymerization at 20 °C.

Based on the polymerization and depolymerization studies (vide supra), a structure–polymerizability–depolymerizability relationship can be established, as shown in Figure 5. Notably, M2 exhibits a high monomer conversion of 91%, coupled with a depolymerization efficiency of 91% for its corresponding polymer. These results indicate that its RSE (4.78 kcal/mol) is well-suited for both polymerization and depolymerization processes. This finding aligns with previous reports on depolymerizable polymers derived from cyclic olefin monomers, which typically have RSE values in the range of 4.7–5.4 kcal/mol.^33,39,43^

### Chemical Recycling of P1 via Functional Group Transformation Strategy

**Figure 6:**
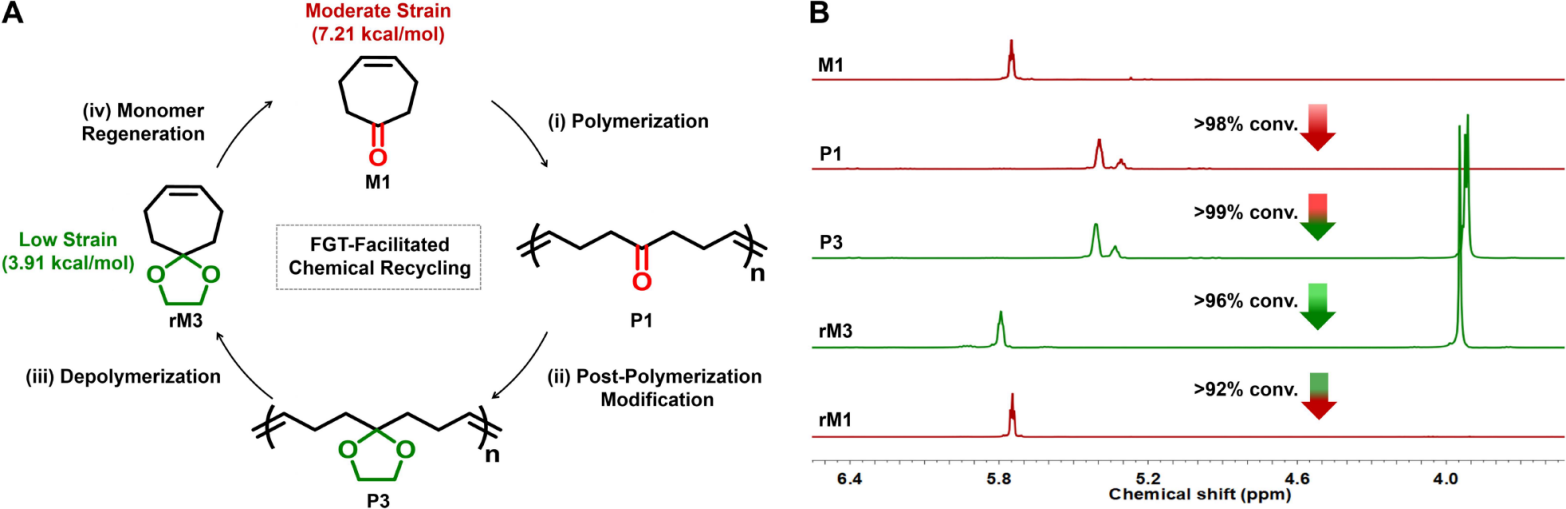
Chemical recycling of P1 via functional group transformation strategy. (A) Four-step chemical recycling process: (i) polymerization of M1; (ii) transformation of P1 to P3; (iii) depolymerization of P3; and (iv) hydrolysis of M3 to regenerate M1. (B) Partial ^1^H NMR spectra of M1, P1, P3, regenerated M3 (rM3), and regenerated M1 (rM1).

Despite the moderate depolymerizability of P1, its ability to transform into highly depolymerizable polymer structures (i.e., P3 and P4) would facilitate the chemical recycling process. In light of this, we leveraged the FGT strategy to chemically recycle P1 back into M1 (Figure 6A). It is worth noting that the reversible and highly efficient nature of ketone-to-acetal chemistry is critical to ensure the effectiveness of this approach.

NMR was used to evaluate the efficiency of each step during the chemical recycling (Figures 6B, S38, and S39). The first step, involving ROMP of M1, led to P1 with more than 98% monomer conversion (Figure 3C). In the second step, the transformation of P1 to P3 was achieved through the reaction of ketone groups with ethylene glycol, using reaction conditions similar to those employed in the conversion of M1 into M3. NMR analysis of the resulting P3 indicates a quantitative conversion of the ketone groups into cyclic acetals alongside the polymer backbone (Figure S38). Depolymerization of P3, synthesized via postpolymerization modification of P1, further yielded M3 with over 96% conversion. Finally, M3 was efficiently hydrolyzed into M1, closing the loop of recycling process (Figure 6B).

### Thermal Properties of Functional Polyheptenamers

**Figure 7:**
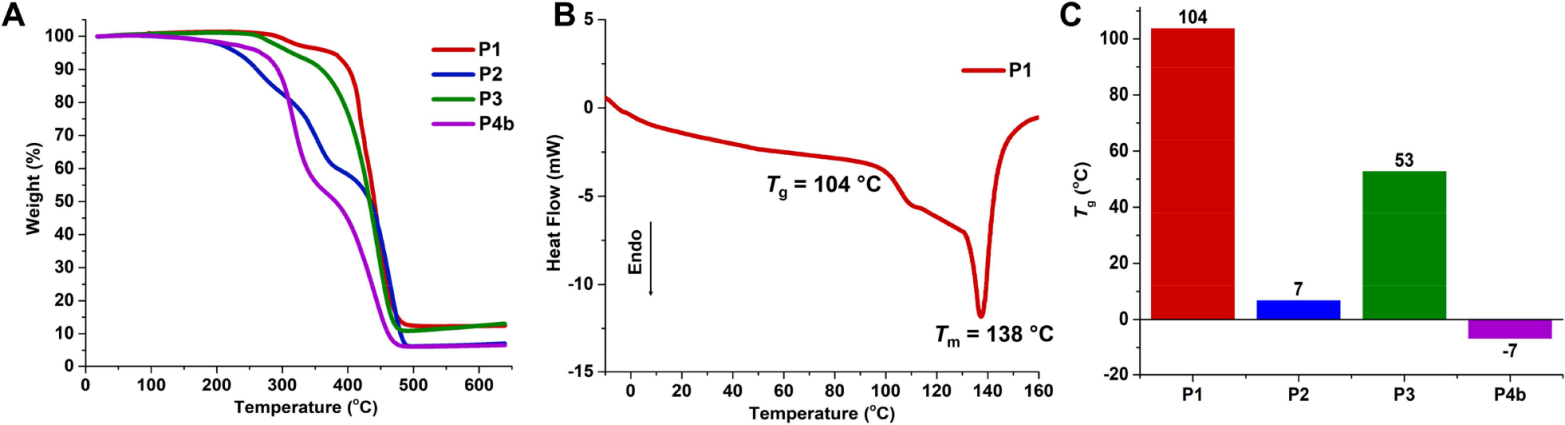
Thermal properties of depolymerizable polyheptenamers. (A) Thermogravimetric analysis of functional polyheptenamers (P1–P4b). (B) Differential scanning calorimetry (DSC) thermogram of P1. (C) Glass transition temperatures of P1–P4b determined by DSC.

To predict the potential of functional polyheptenamers for industrial applications, we further assessed their thermal properties (Figures 3C and 7). Thermogravimetric analysis revealed the high thermal stability of P1–P4b, with their decomposition temperatures at 5% weight loss (*T*_d_) ranging from 226 to 381 °C (Figure 7A). The relatively lower thermal stability of P2–P4b compared to P1 can be ascribed to their thermally labile side-chain groups: ester (P2) and acetals (P3 and P4b).

Glass transition temperatures (*T*_g_) of the polymers were further evaluated by differential scanning calorimetry (DSC) (Figures 7B,C and S40–S43). Based on the DSC thermogram of P1, a high *T*_g_ of 104 °C and a *T*_m_ of 138 °C were observed, suggesting the semicrystalline nature of the polyketone structure and restricted chain mobility arising from dipole–dipole interactions among ketone groups (Figure 7B). By contrast, the *T*_g_ values of P2–P4b, which bear large side chains, were significantly lower than that of P1 (Figure 7C). The wide range of glass transition temperatures (−7 to 104 °C) exhibited by functional polyheptenamers illustrates their potential for applications such as plastics and elastomers.

## Conclusions

In summary, we demonstrate a class of chemically recyclable polyolefins based on cycloheptene-derived monomers with a wide range of RSEs (3.8–7.2 kcal/mol). The library of functional monomers and polymers enabled the establishment of a structure–polymerizability–depolymerizability relationship that elucidates the role of RSE in both polymerization and depolymerization. A functional group transformation approach was harnessed to transform polymers with varying propensities to depolymerize, not only facilitating the chemical recycling of moderately depolymerizable polymers, but also providing access to highly depolymerizable polyolefins that are difficult to synthesize by ROMP of low-strain monomers directly. Moreover, these functional polymers display a broad range of glass transition temperatures, demonstrating their potential for various applications. Given the promise of depolymerizable polymers in circular polymer economy, we envision that the functional polyheptenamers developed in this study will lead to a new class of sustainable and eco-friendly polymer materials with highly tunable properties. The functional group transformation approach provides a new strategy to expand the scope of cyclic olefin monomers for the development of chemically recyclable polyolefin materials with diverse structures.

## Supporting Information Available

General experimental procedure, NMR spectra of monomers and polymers, SEC and DSC analyses of polymer materials, and computational details (PDF)
