# Supplementary material for: Functional Group Transformation Approach to Chemically Recyclable Polymers from Ultra-Low to Moderate Strain Monomers
Source: Macromolecules. 2025 Apr 11;58(8):3898–905. doi: 10.1021/acs.macromol.4c03248 (PMC12020419; doi:10.1021/acs.macromol.4c03248)
Supplement: Supplementary file 1 — ma4c03248_si_001.pdf [file ma4c03248_si_001.pdf]

Supporting Information

for

A Functional Group Transformation Approach to Chemically Recyclable Polymers from Ultra-Low to Moderate Strain Monomers

Tarek Ibrahim, Kaia Kendzulak, Angelo Ritacco, Melanie Monetti, and Hao Sun*

Department of Chemistry and Chemical & Biomedical Engineering, Tagliatela College of Engineering, University of New Haven, West Haven, Connecticut 06516, USA

Corresponding to: Dr. Hao Sun, Email: hasun@newhaven.edu

Table of Contents

1. Materials 2
2. Instrumentation 2
3. Experimental Methods 3
  3.1. Computational Study 3
  3.2. Synthesis of M2 3
  3.3. Synthesis of M3 4
  3.4. Synthesis of M4 5
  3.5. Polymerization Protocol 5
  3.6. Depolymerization Ptotocol 6
  3.7. Transformation of P1 to P3 6
  3.8. Transformation of P1 to P4 7
  3.9. Hydrolysis of M3 7
4. Supporting Tables and Figures 8
5. Optimized Geometries of Monomers and Their Ring-Opened Structures from DFT Calculations 29
6. References 38

1. Materials

4-Bromo-1-butene (97%), ethyl formate (97%), magnesium (>99.5%), Jones reagent, acetic anhydride (>99%), ethylene glycol (anhydrous, 99.8%), pinacol (98%), sodium borohydride (99%), methanol (>99.8%) ethyl vinyl ether (99%), Toluene-4-sulfonic acid monohydrate (*p*-TsOH), triethyl orthoformate (TEOF) (anhydrous, 98 %), triethylamine (TEA) (>99.5%), 4-(Dimethylamino)pyridine (DMAP) (>99%), $CDCl_3$ (99.8 atom % D), CD_2Cl_2 (99.5 atom % D), anhydrous dichloromethane (DCM, 99.8%), Grubbs' catalyst 2nd generation (M204) were purchased from Sigma Aldrich and used without purification. 4-Cyclohepten-1-one (M1) was synthesized accoding to a previous study.[1]

2. Instrumentation

Nuclear Magnetic Resonance (NMR): ^{1}H NMR spectra were recorded on a Bruker spectrometer (400 MHz) in $CDCl_3$ or CD_2Cl_2. Chemical shifts are given in ppm downfield from tetramethylsilane (TMS).

Gas Chromatography-Mass Spectrometry (GC-MS): Gas chromatography-mass spectrometry (GC-MS) analyses were performed on a Shimadzu 2010 series GC system equipped with a Shimadzu QP 2010s mass selective detector, an AOC-20i autosampler, and a Quadrex column (007 MPS5 - 25 m -0.25 mm – I.D. 0.5 μm).

Size Exclusion Chromatography (SEC): The molecular weight and polydispersity of synthetic polymers were determined by a size exclusion chromatography (SEC) system (TOSOH EcoSEC HLC-8320) equipped with a set of Phenomenex Phenogel 5μ, 1K-75K, 300 x 7.80 mm in series with a Phenomex Phenogel 5μ, 10K-1000K, 300 x 7.80 mm columns following a guard column and two detectors including a RI detector and a UV detector. The measurements were performed using HPLC-grade THF as the eluent at a flow rate of 0.5 mL/min at 35 °C and a series of polystyrene standards for the calibration of the columns.

Differential Scanning Calorimetry (DSC): Differential scanning calorimetry (DSC) measurements were performed using a Guangdong Newgoer DSC-300C system under a nitrogen gas flow (100 mL/min). Two thermal cycles with heating and cooling rates of 10 °C/min

were performed. The melting and crystalization temperatures were obtained from the second heating and cooling scans after removing the thermal history of polymers.

Thermogravimetric Analysis (TGA): Thermogravimetric analysis (TGA) was performed using a TA SDT Q600 system under a nitrogen gas flow (100 mL/min) with a heating rate of 10 °C/min. The temperature range for the analysis extended from ambient temperature to 650 °C.

3. Experimental Methods

3.1. Computational Study

The ring strain energies of cyclic olefin monomers were calculated using density functional theory (DFT) with Spartan software. To account for the various conformations available in both the monomers and their ring-opened products (i.e., nonadiene derivatives), an exhaustive conformer search was performed at the AM1 level of theory. The geometries and energies of the resulting conformers were then optimized at the B3LYP/6-31G(d) level of theory in the gas phase. By retaining conformers that contributed to more than 95% of the Boltzmann population, the final ensembles consisted of 40 to 80 conformers for the nonadiene derivatives and 2 to 5 conformers for the cycloheptene-based monomers. All energy values used in the calculations were Boltzmann-weighted averages of these conformers. The enthalpy changes (ΔH) were estimated as the enthalpy difference between the ring-opened products and the total enthalpy of the isolated reactants (monomer + ethylene).

3.2. Synthesis of M2

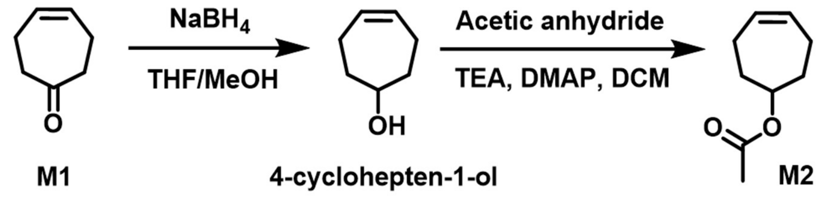

Synthesis of 4-cyclohepten-1-ol: To a 25 mL round-bottom flask, M1 (1.0 g, 9.1mmol) was dissolved in 9 mL mixture of THF/MeOH (v/v = 8:1). Separately, $NaBH_4$ (0.85 g, 22 mmol) was dissolved in 8 mL of THF and cooled to 0 °C. The M1 solution was then added dropwise to the

cold $NaBH_4$ solution under stirring. The resulting mixture was stirred for an additional 10 min at 0 °C and then allowed to warm up to room temperature. The reaction mixture was stirred at room temperature for 24 h. The progress of the reaction was monitored using TLC. After confirming the completion of reaction, the mixture was quenched with 10 mL of water. The aqueous layer was extracted with ethyl acetate (5 x 10 mL). The combined organic layers were washed with brine (10 mL) and dried over anhydrous sodium sulfate (Na_2SO_4). After filtering off the drying agent, the filtrate was concentrated under reduced pressure to obtain 4-cyclohepten-1-ol as a light yellow oil (0.85 g, 83%).

Synthesis of M2: In a dry flask, 4-cyclohepten-1-ol (1.0 g, 8.9 mmol), triethylamine (1.86 mL, 13.4 mmol), and DMAP (163 mg, 1.34 mmol) were suspended in 30 mL of dry DCM. A solution of acetic anhydride (1.26 mL, 13.4 mmol) in 14 mL of dry DCM was added dropwise to the solution of 4-cyclohepten-1-ol at 0 °C. The reaction mixture was stirred at 0 °C for 3 h. After that, the mixture was allowed to warm up to room temperature and stirred for another 12 h. The DCM solution was washed sequentially with saturated sodium bicarbonate ($NaHCO_3$) solution, 1 M HCl solution, and brine. The organic phase was dried over Na_2SO_4, and concentrated under reduced pressure. The crude product was purified by flash column chromatography (4:6 EtOAc/Hexanes) to yield M2 as a light yellow oil (0.91 g, 66%).

3.3. Synthesis of M3

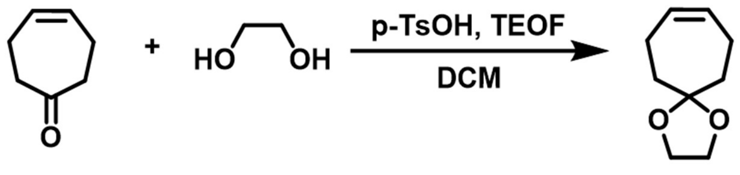

M1 M3

A round bottom flask equipped with a stir bar and reflux condenser was charged with anhydrous DCM (22 mL), M1 (0.50 g, 4.5 mmol), ethylene glycol (1.1 mL, 20 mmol), *p*-TsOH (0.080 g, 0.45 mmol), and triethyl orthoformate (2.6 mL, 16 mmol). The reaction mixture was then heated to reflux in an oil bath for 12 h. After the reaction, the solution was cooled and washed sequentially with water (15 mL) and brine (15 mL). The organic layer was then dried

over Na_2SO_4 and concentrated under reduced pressure. The crude product was purified by column chromatography (4:6 EtOAc/Hexanes) to yield M3 as a colorless liquid (0.55 g, 79%).

3.4. Synthesis of M4

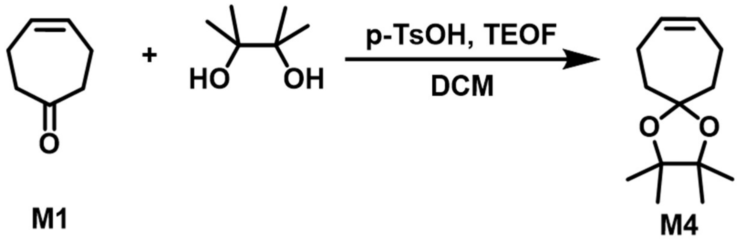

A round bottom flask equipped with a stirring bar and reflux condenser was charged with anhydrous DCM (22 mL), M1 (0.50 g, 4.5 mmol), pinacol (2.4 g, 20 mmol), *p*-TsOH (0.080 g, 0.45 mmol), and triethyl orthoformate (2.6 mL, 16 mmol) . The reaction mixture was then heated to reflux in an oil bath for 12 h. After the reaction, the solution was cooled and washed sequentially with water (15 mL) and brine (15 mL). The organic layer was then dried over Na2SO4 and concentrated under reduced pressure. The crude product was purified by column chromatography (3:7 EtOAc/Hexanes) to yield M4 as a colorless liquid (0.71 g, 74%).

3.5. Polymerization Protocol

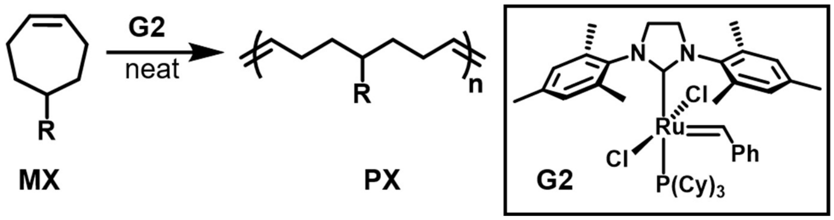

In a typical procedure for the bulk ROMP of M1 at room temperature, M1 (0.50 g, 4.5 mmol, 120 equiv.) was added in 2 mL vial, and degassed by argon gas for 10 min. Catalyst G2 (32 mg, 0.038 mmol, 1.0 equiv.) was then quickly added to the monomer under stirring. The reaction mixture was stirred at room temperature for 12 h. The polymerization was quenched with excess ethyl vinyl ether (355 µL, 3.80 mmol, 100 equiv.) under stirring. After the quenching process for 20 min, 10 µL of the solution was taken and diluted in 0.5 mL of $CDCl_3$ for NMR

analysis to estimate the monomer conversion. The remaining solution was precipitated into cold methanol to obtain the polymer product.

3.6. Depolymerization Ptotocol

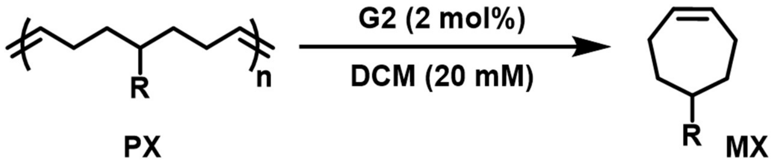

In a typical procedure for the RCMD of functional polyheptenamers (e.g., P2) in a dilute solution (20 mM of olefins) at 20 °C, P2 (15.7 mg, 0.104 mmol of olefins, 50 equiv.) and G2 (1.8 mg, 0.00208 mmol, 1.0 equiv.) were dissolved in dry DCM (5.2 mL) and then degassed by argon gas. The reaction mixture was stirred at 20 °C for 12 h. The depolymerization was then quenched with excess ethyl vinyl ether (20 µL, 0.208 mmol, 100 equiv.). After the quenching process for 20 min, the solution was analyzed by NMR to estimate the depolymerization efficiency.

3.7. Transformation of P1 to P3

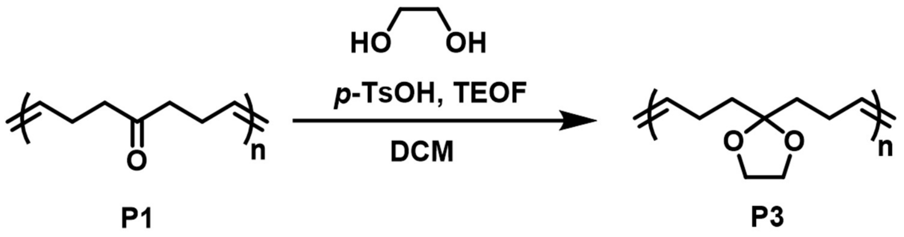

A 20 mL round bottom flask equipped with a stirring bar and reflux condenser was charged with anhydrous DCM (8.8 mL), P1 (200 mg, 1.8 mmol), ethylene glycol (0.44 mL, 8.0 mmol), *p*-TsOH (32 mg, 0.18 mmol), and triethyl orthoformate (1.04 mL, 6.40 mmol). The reaction mixture was stirred at 35 °C for 24 h. After the reaction, the solution was cooled and washed sequentially with water (6 mL) and brine (6 mL). The organic layer was concentrated under reduced pressure, and then precipitated into cold methanol to yield the polymer product.

3.8. Transformation of P1 to P4

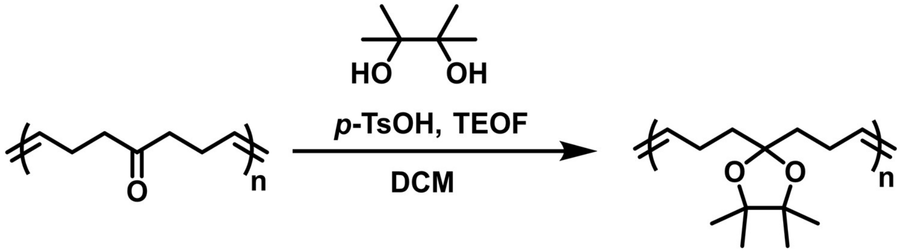

P1 P4

A 20 mL round bottom flask equipped with a stirring bar and reflux condenser was charged with anhydrous DCM (8.8 mL), P1 (200 mg, 1.8 mmol), pinacol (0.96 g, 8.0 mmol), *p*-TsOH (32 mg, 0.18 mmol), and triethyl orthoformate (1.04 mL, 6.40 mmol). The reaction mixture was stirred at 35 °C for 24 h. After the reaction, the solution was cooled and washed sequentially with water (6 mL) and brine (6 mL). The organic layer was concentrated under reduced pressure, and then precipitated into cold methanol to yield the polymer product.

3.9. Hydrolysis of M3

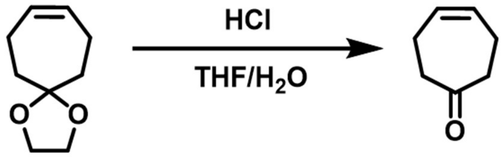

M3 M1

In a typical hydrolysis protocol, M3 (0.20 g, 1.3 mmol) and 7 mL of THF were added in a 20 mL vial. 3 mL of 1 M HCl aqueous solution was added dropwise into the M3 solution. The reaction mixture was stirred at room temperature for 24 h. After the reaction, the solution was diluted with ethyl acetate (20 mL) and washed sequentially with saturated $NaHCO_3$ solution and brine. The organic layers were combined and dried over Na_2SO_4 and concentrated under reduced pressure. The crude product was purified by column chromatography to yield M1 as a colorless liquid (0.13 g, 92%).

4. Supporting Tables and Figures

Table S1. Polymerization Results

**Entry[a]**	**Monomer Type**	**Solvent & Concentration**	**Temperature (°C)**	**Monomer Conversion[b] (%)**
**1**	M1	DCM (0.5 M)	20	64
**2**	M1	DCM (0.5 M)	30	58
**3**	M1	DCM (0.5 M)	40	55
**4**	M1	Neat	20	98
**5**	M2	Neat	20	91
**6**	M3	Neat	20	81
**7**	M4	Neat	20	13

[a)]Polymerizations were carried out with a monomer-to-catalyst feed ratio of 120 under an argon atmosphere for 12 h; [b)]The monomer conversions were determined by ^{1}H NMR spectroscopy.

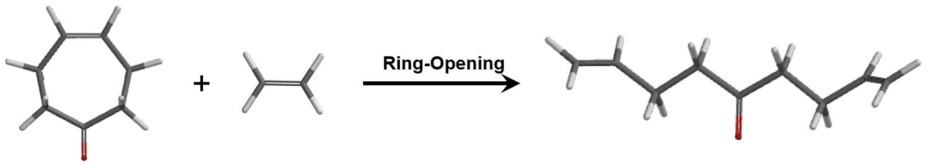

M1 **Ethylene** **Ring-Opened Product**

**Chemical Structure**	**Enthalpy (Hartree)**
4-Cycloheptenone (M1)	-347.802435
Ethylene	-78.5322597
Ring-Opened Product	-426.346188

RSE = -ΔH = 426.346188 - 426.334695 = 0.011493 Hartree = 7.21 kcal/mol

Figure S1. DFT calculation of ring strain energy (RSE) of M1. A B3LYP/6-31G* level of theory was applied for the geometry optimization and energy calculation of conformers in vacuum. The energies were calculated as Boltzmann-weighted averages of conformers contributing to more than 95% of the population.

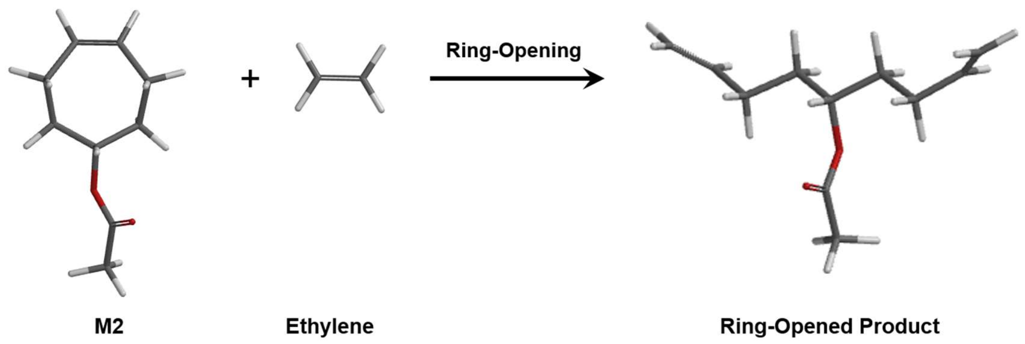

Chemical Structure	Enthalpy (Hartree)
M2	-501.607807
Ethylene	-78.5322597
Ring-Opened Product	-580.147685

RSE = -ΔH = 580.147685 - 580.140067 = 0.007618 Hartree = 4.78 kcal/mol

Figure S2. DFT calculation of ring strain energy (RSE) of M2. A B3LYP/6-31G* level of theory was applied for the geometry optimization and energy calculation of conformers in vacuum. The energies were calculated as Boltzmann-weighted averages of conformers contributing to more than 95% of the population.

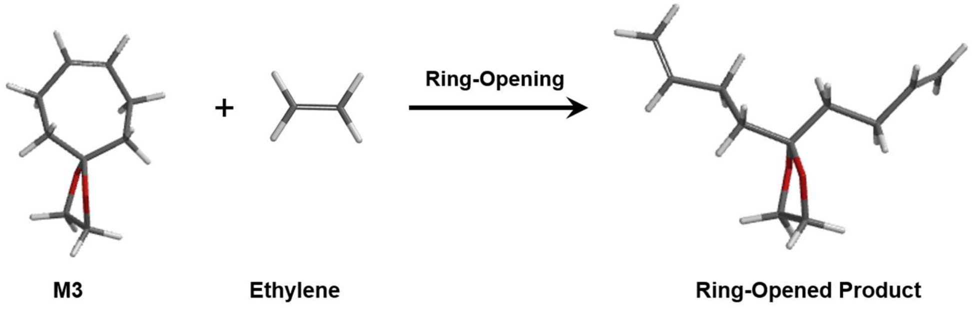

Chemical Structure	Enthalpy (Hartree)
M3	-501.570135
Ethylene	-78.5322597
Ring-Opened Product	-580.108631

RSE = -ΔH = 580.108631 - 580.102395 = 0.006236 Hartree = 3.91 kcal/mol

Figure S3. DFT calculation of ring strain energy (RSE) of M3. A B3LYP/6-31G* level of theory was applied for the geometry optimization and energy calculation of conformers in vacuum. The energies were calculated as Boltzmann-weighted averages of conformers contributing to more than 95% of the population.

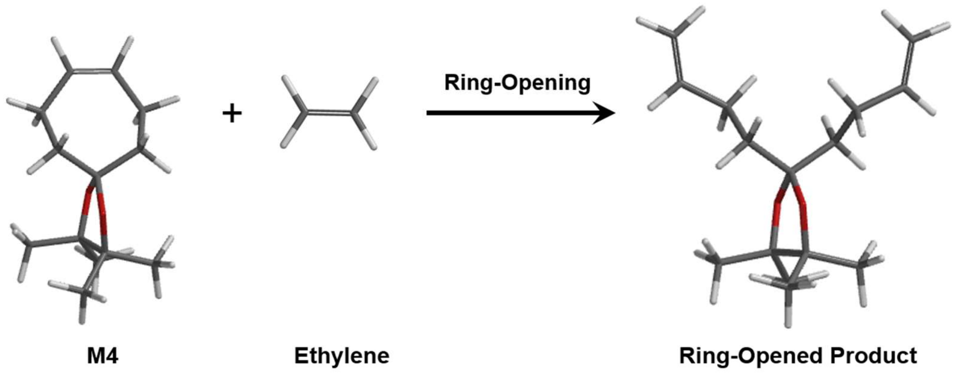

Chemical Structure	Enthalpy (Hartree)
M4	-658.719833
Ethylene	-78.5322597
Ring-Opened Product	-737.258228

RSE = -ΔH = 737.258228 - 737.252093 = 0.006135 Hartree = 3.85 kcal/mol

Figure S4. DFT calculation of ring strain energy (RSE) of M4. A B3LYP/6-31G* level of theory was applied for the geometry optimization and energy calculation of conformers in vacuum. The energies were calculated as Boltzmann-weighted averages of conformers contributing to more than 95% of the population.

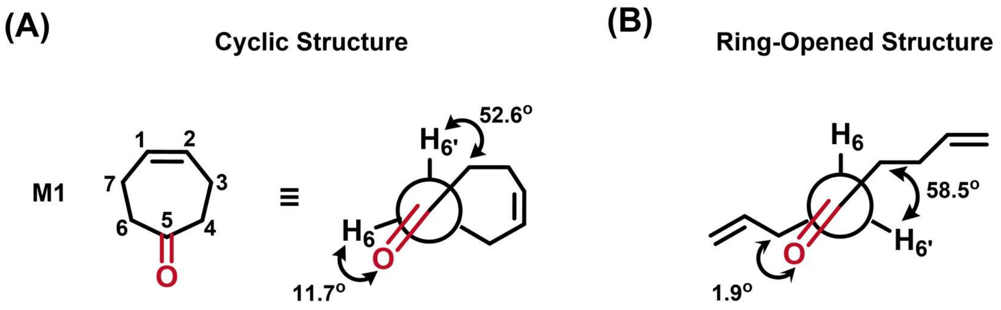

Figure S5. Newman projections along the C5-C6 bond for M1 (A), and the ring-opened acyclic structure (B).

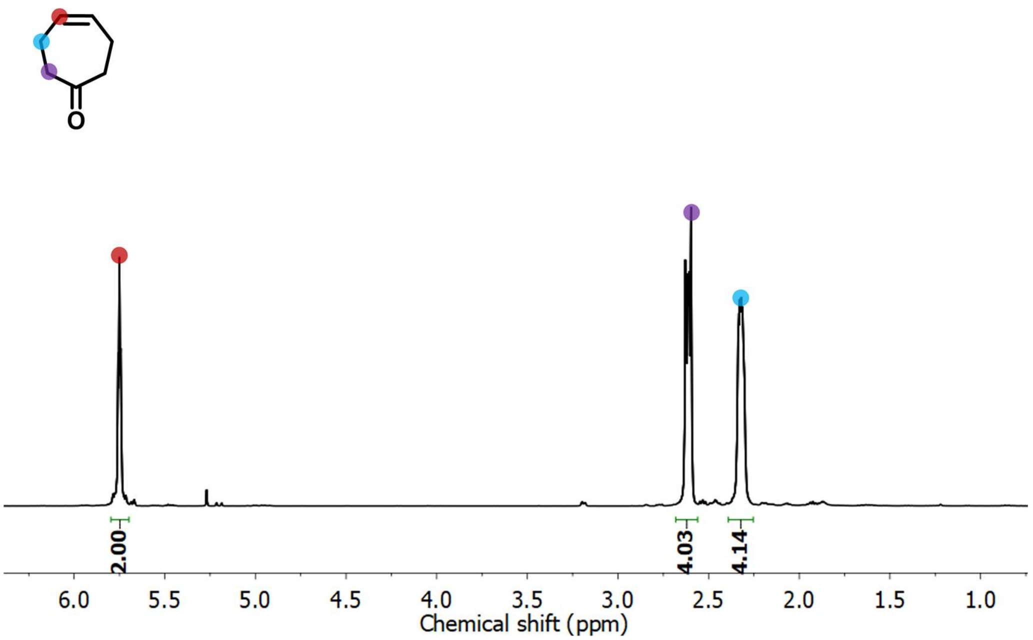

Figure S6. ^{1}H NMR spectrum of M1 in $CDCl_3$ at room temperature.

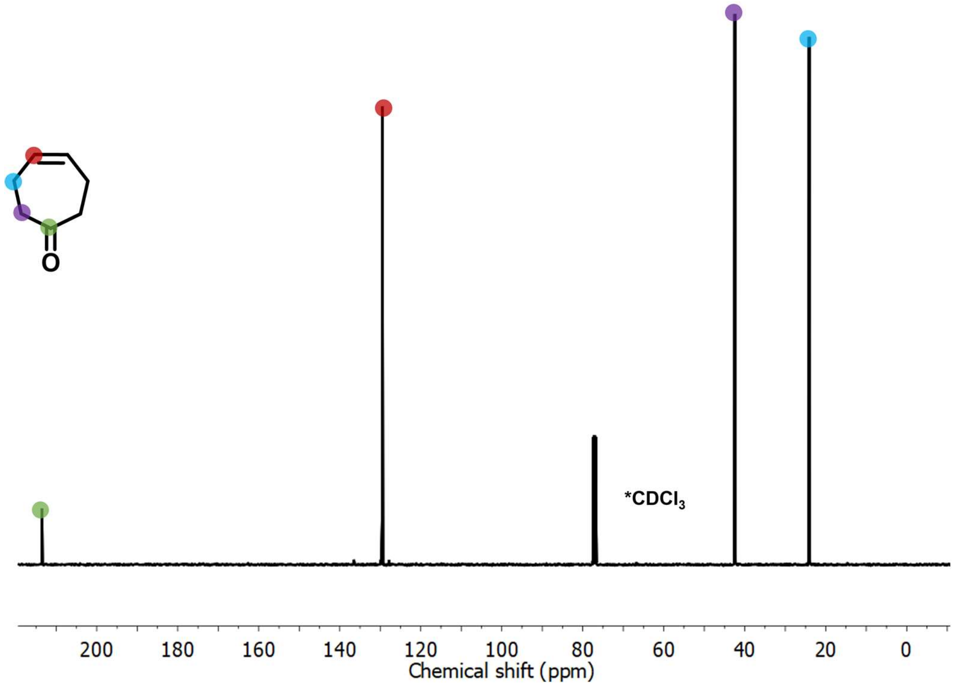

Figure S7. ^{13}C NMR spectrum of M1 in $CDCl_3$ at room temperature.

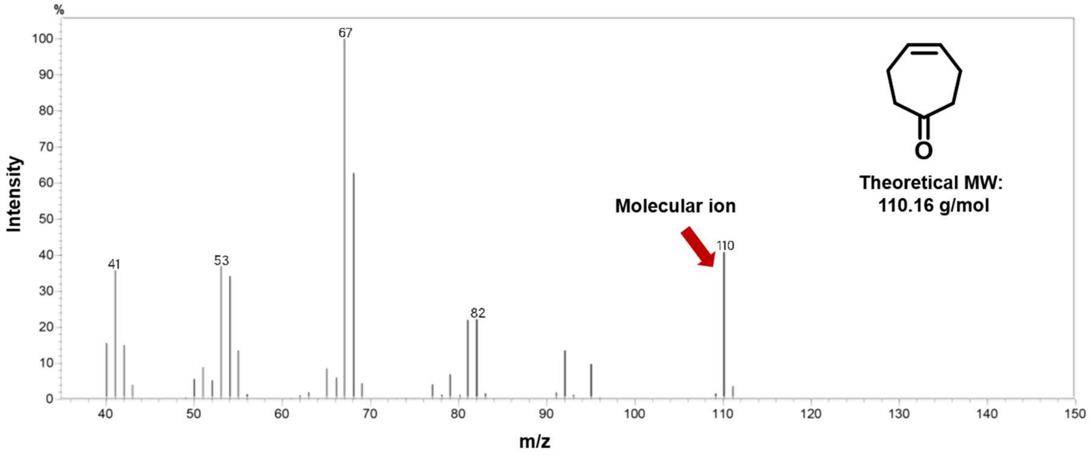

Figure S8. Electron impact mass spectrum of M1.

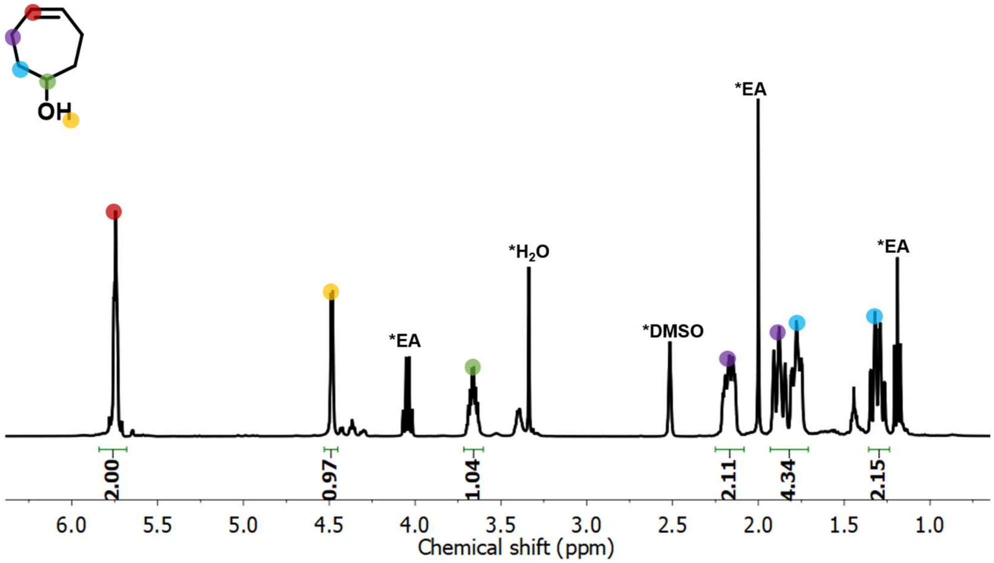

Figure S9. ^1H NMR spectrum of 4-cyclohepten-1-ol in $CDCl_3$ at room temperature.

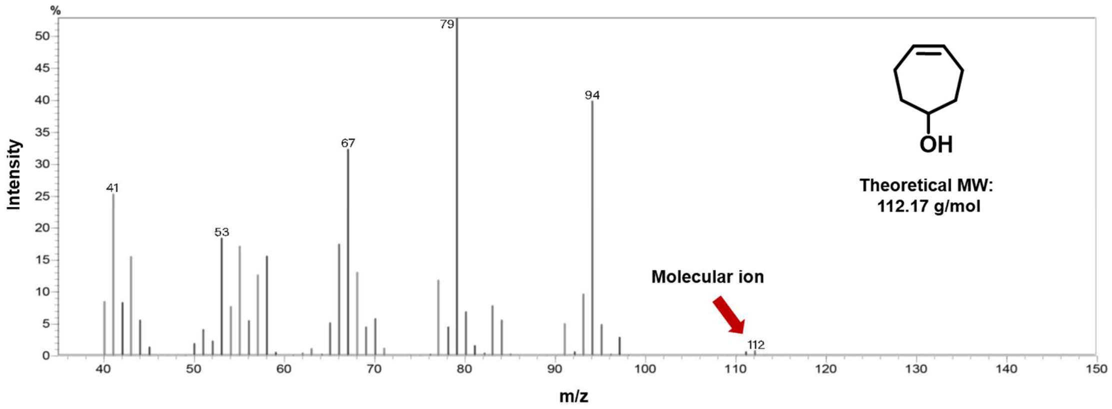

Figure S10. Electron impact mass spectrum of 4-cyclohepten-1-ol.

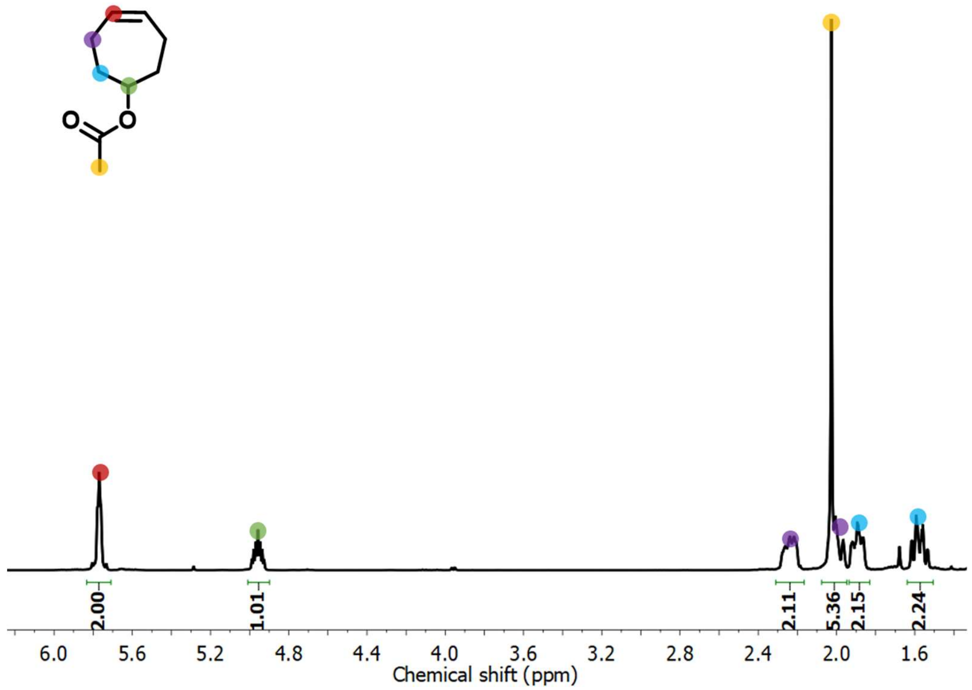

Figure S11. ^{1}H NMR spectrum of M2 in $CDCl_3$ at room temperature.

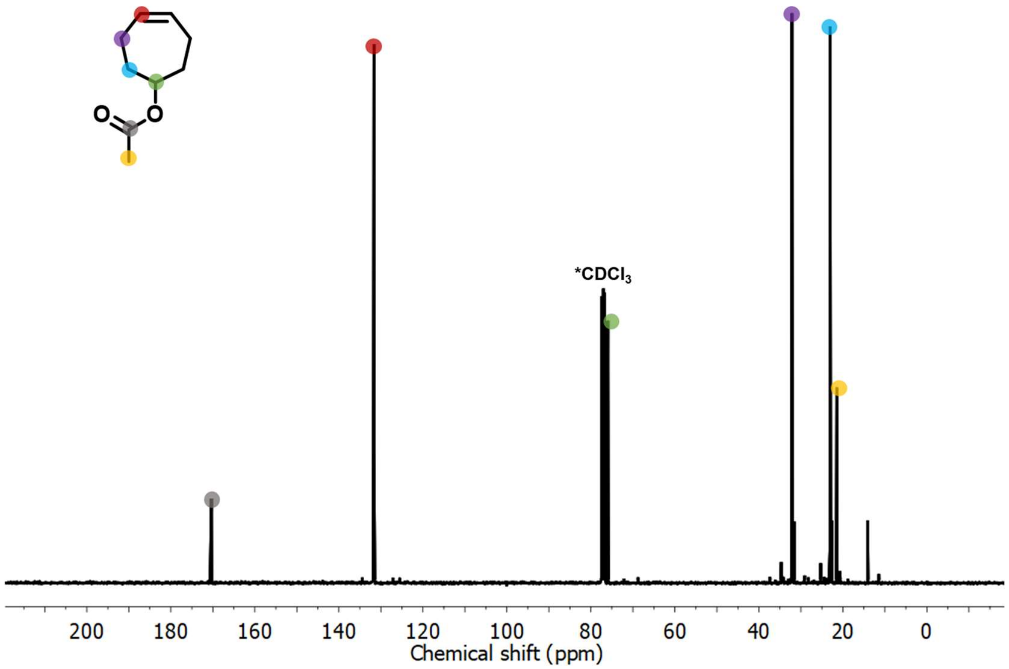

Figure S12. ^{13}C NMR spectrum of M2 in $CDCl_3$ at room temperature.

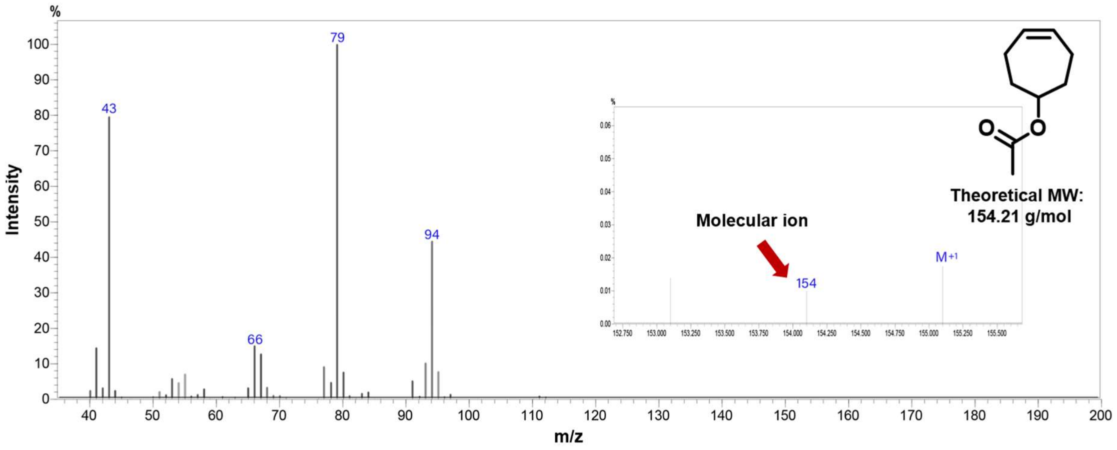

Figure S13. Electron impact mass spectrum of M2.

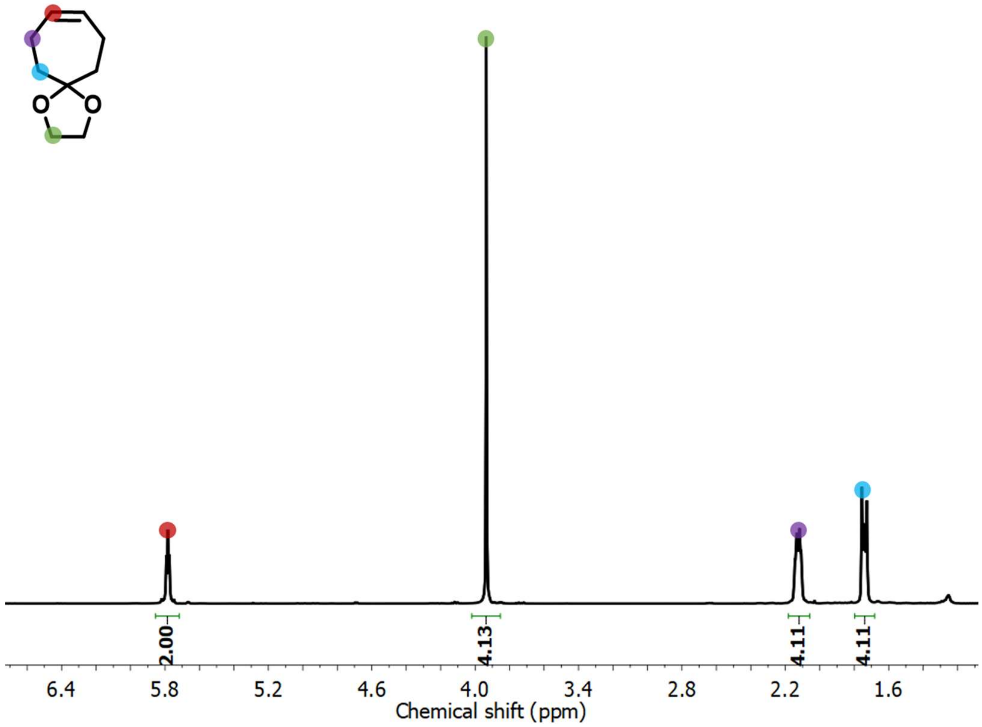

Figure S14. ^{1}H NMR spectrum of M3 in $CDCl_3$ at room temperature.

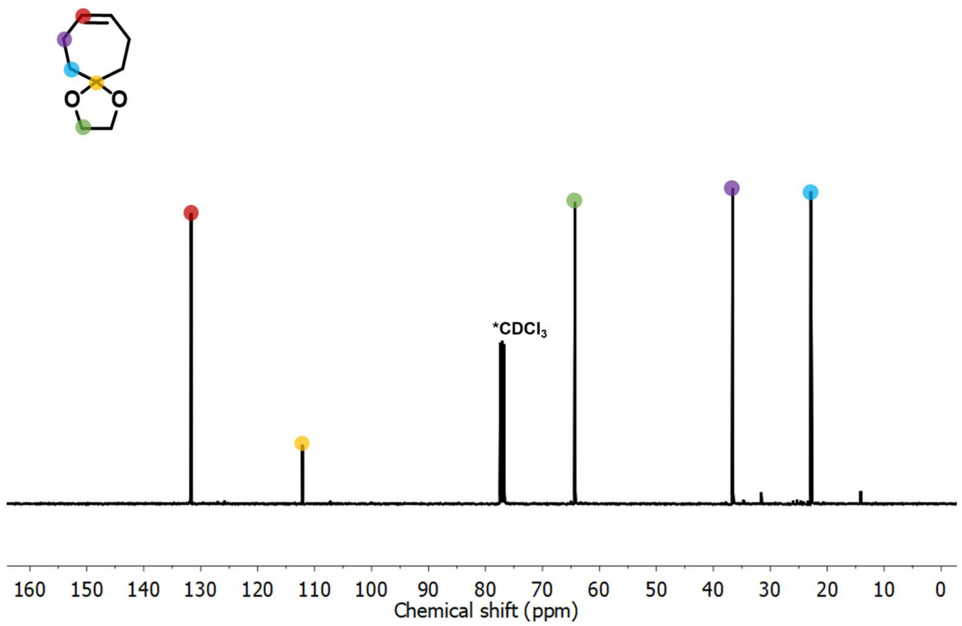

Figure S15. ^{13}C NMR spectrum of M3 in $CDCl_3$ at room temperature.

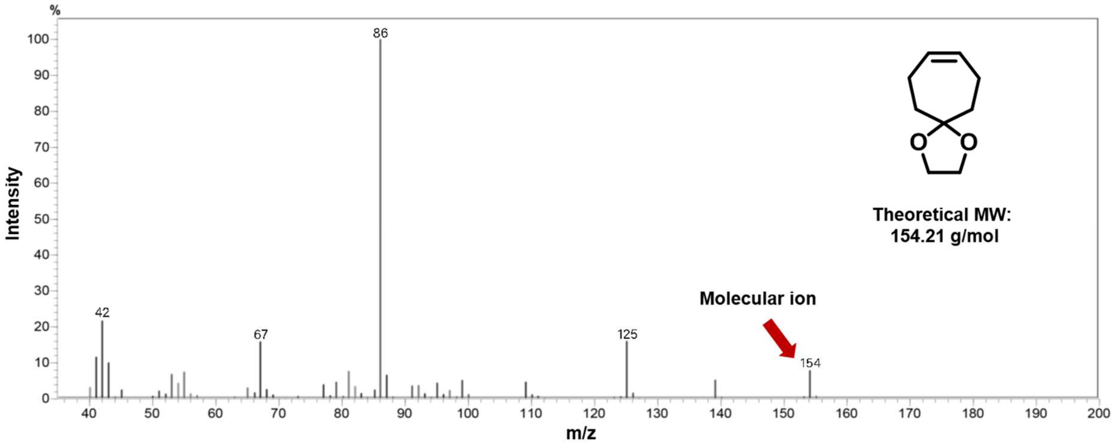

Figure S16. Electron impact mass spectrum of M3.

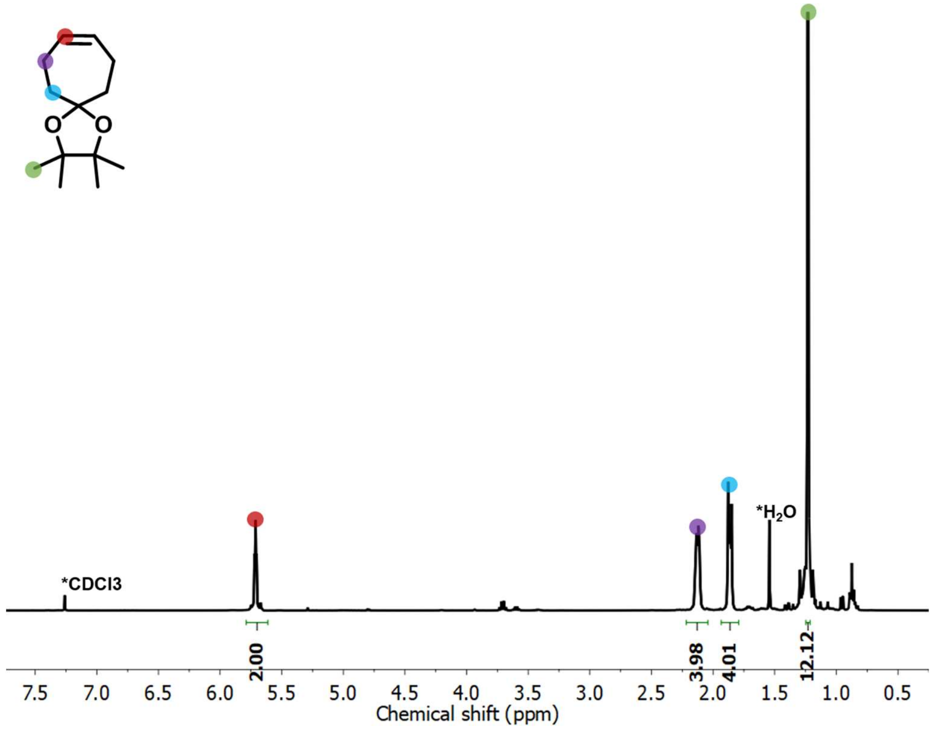

Figure S17. ^{1}H NMR spectrum of M4 in $CDCl_3$ at room temperature.

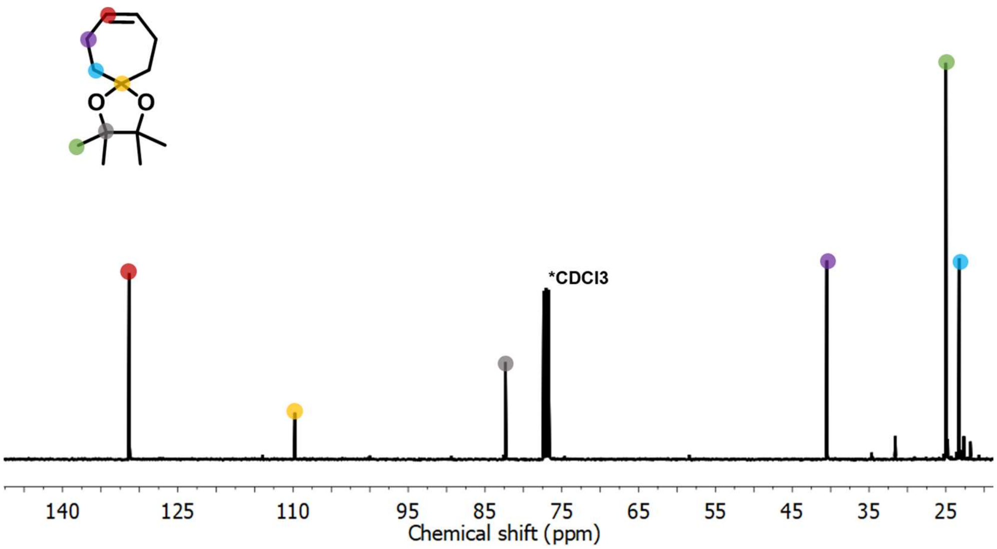

Figure S18. ^{13}C NMR spectrum of M4 in $CDCl_3$ at room temperature.

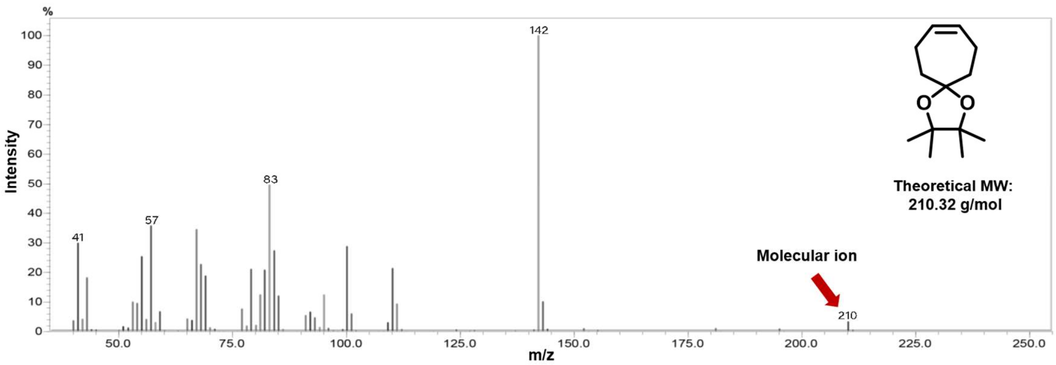

Figure S19. Electron impact mass spectrum of M4.

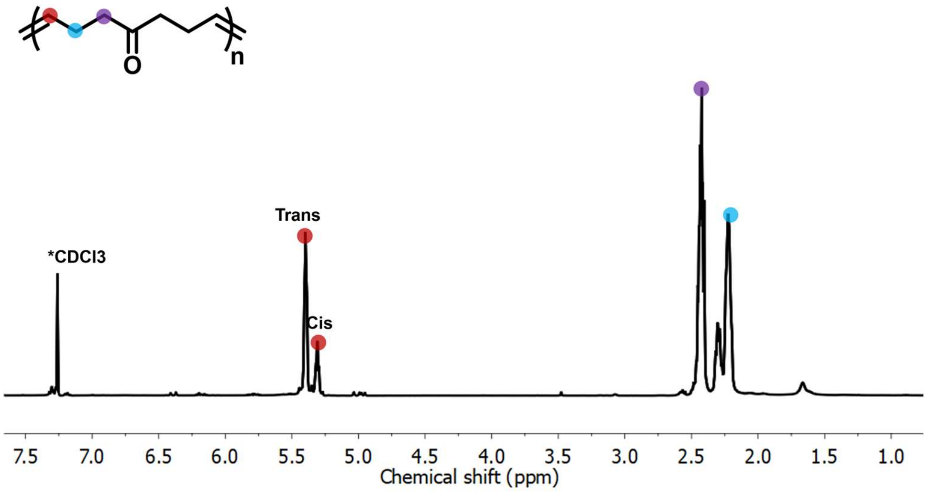

Figure S20. ^{1}H NMR spectrum of P1 in $CDCl_3$ at room temperature.

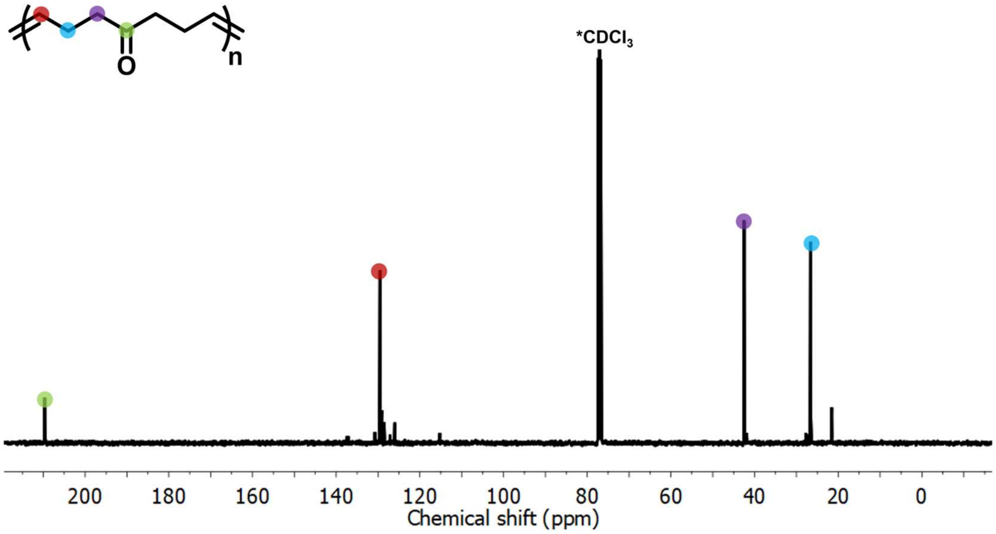

Figure S21. ^{13}C NMR spectrum of P1 in $CDCl_3$ at room temperature.

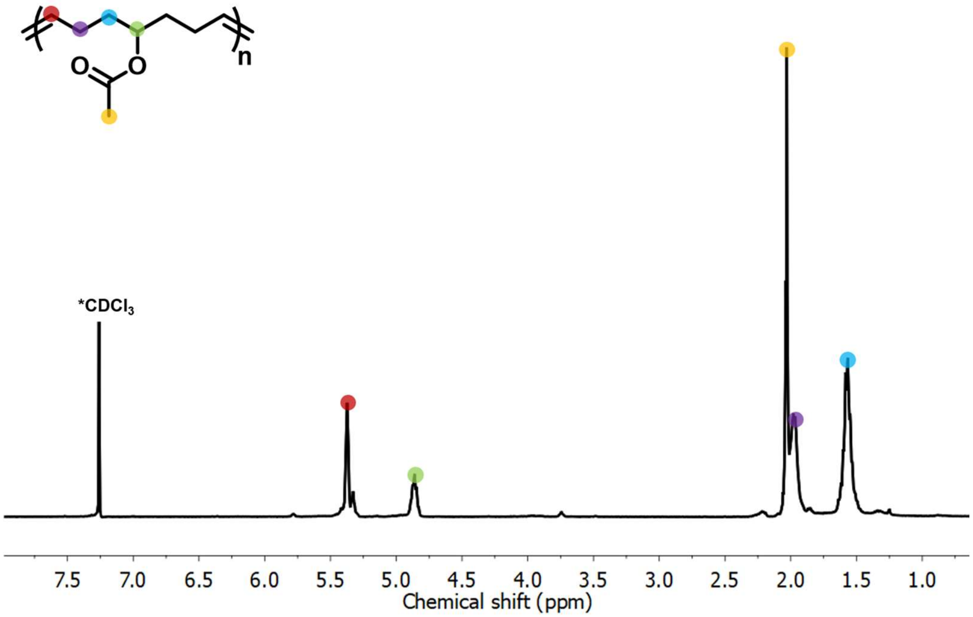

Figure S22. ^{1}H NMR spectrum of P2 in $CDCl_3$ at room temperature.

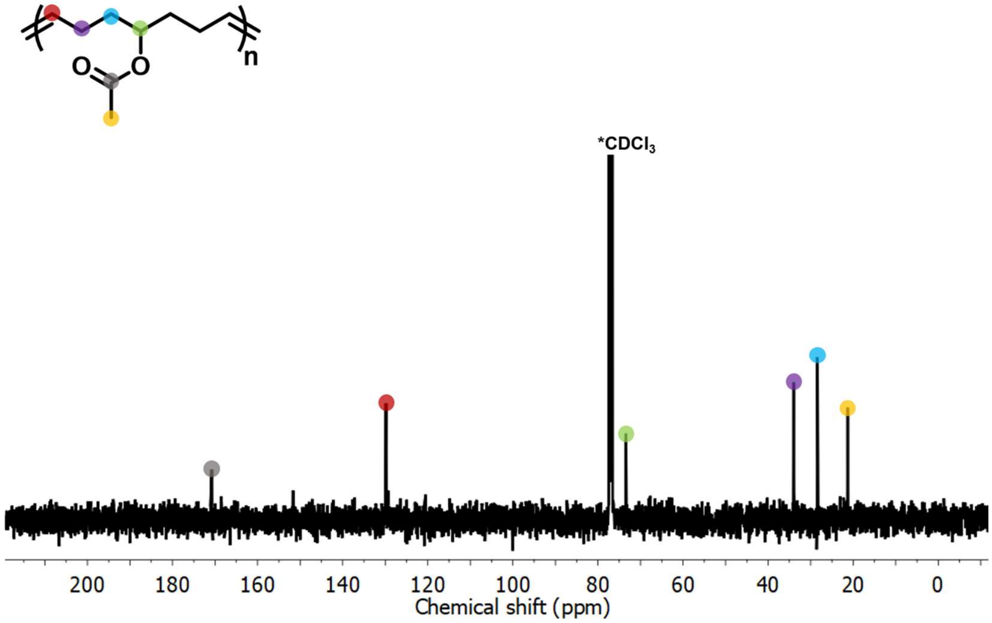

Figure S23. ^{13}C NMR spectrum of P2 in $CDCl_3$ at room temperature.

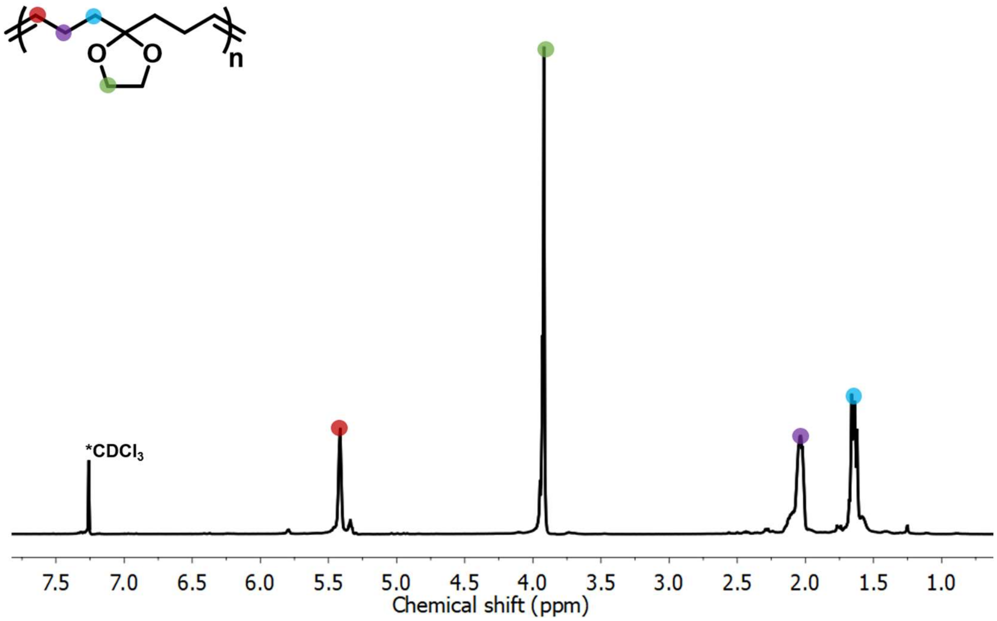

Figure S24. ^{1}H NMR spectrum of P3 in $CDCl_3$ at room temperature.

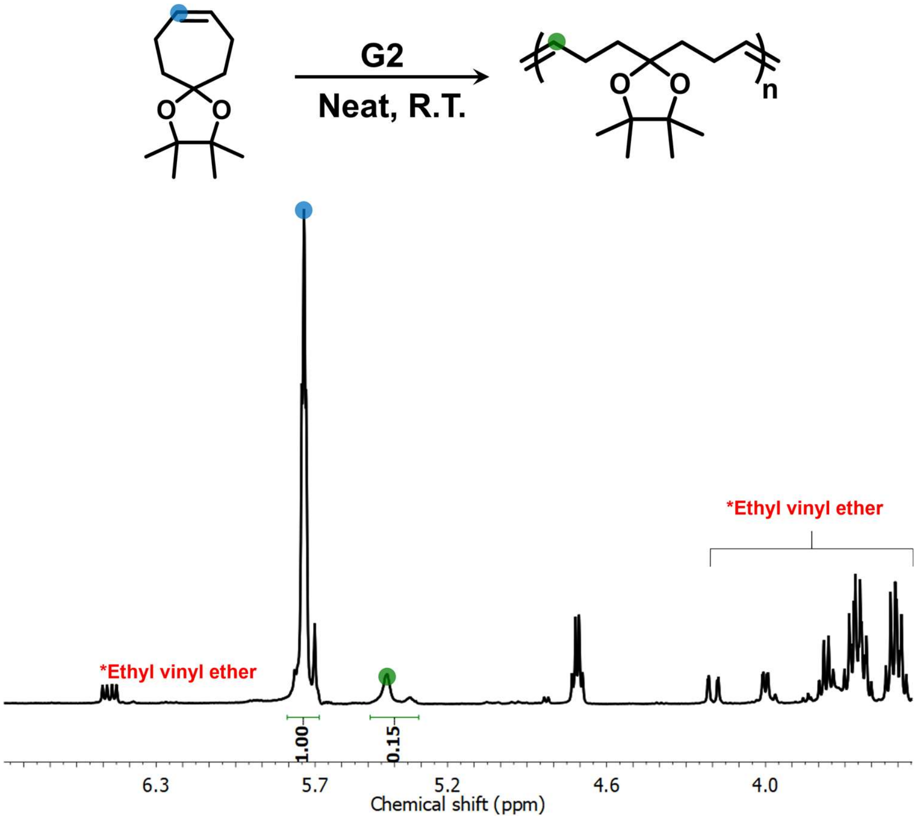

Figure S25. Partial ^{1}H NMR spectrum of the polymerization mixture. The ratio of M4 to G2 was 120. The neat polymerization of M4 was conducted for 12 hours and quenched with ethyl vinyl ether, then diluted in $CDCl_3$ for NMR analysis. Only 13% of M4 was converted to P4a, as determined by comparing the olefin signals of the monomer (highlighted in blue) and the polymer (highlighted in green).

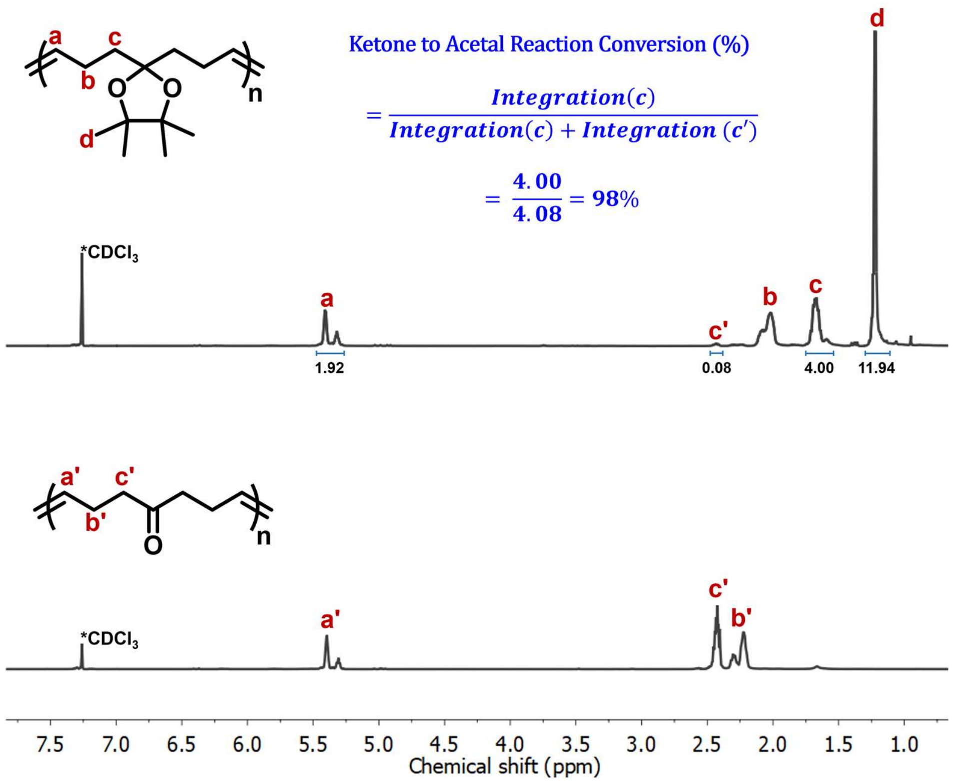

Figure S26. ^{1}H NMR spectra of P4b and P1 in $CDCl_3$ at room temperature. P4b was synthesized *via* functional group transformation of P1 (see section 3.8). The conversion of the ketone to the acetal groups was estimated by comparing the integration of methylene protons (c) in P4b with the methylene protons (c') in the residual P1 repeating units.

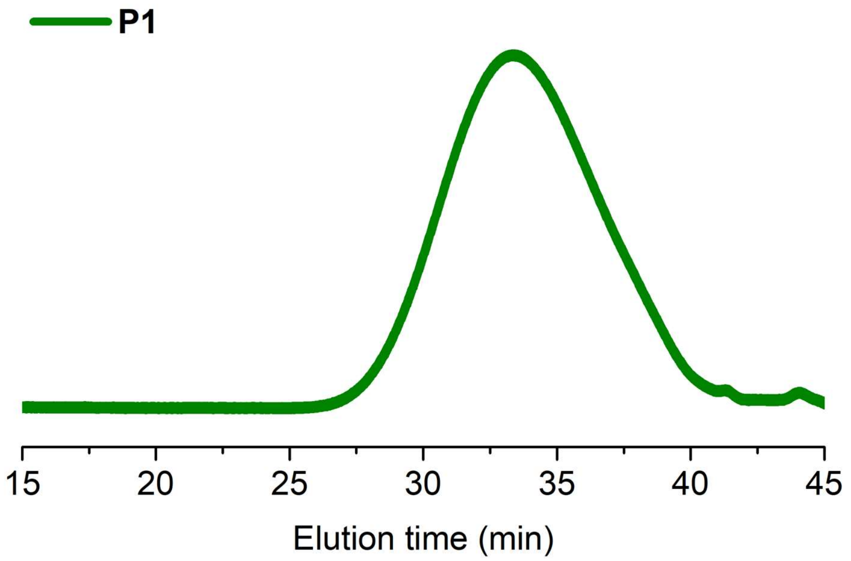

Figure S27. Size exclusion chromatography trace of P1.

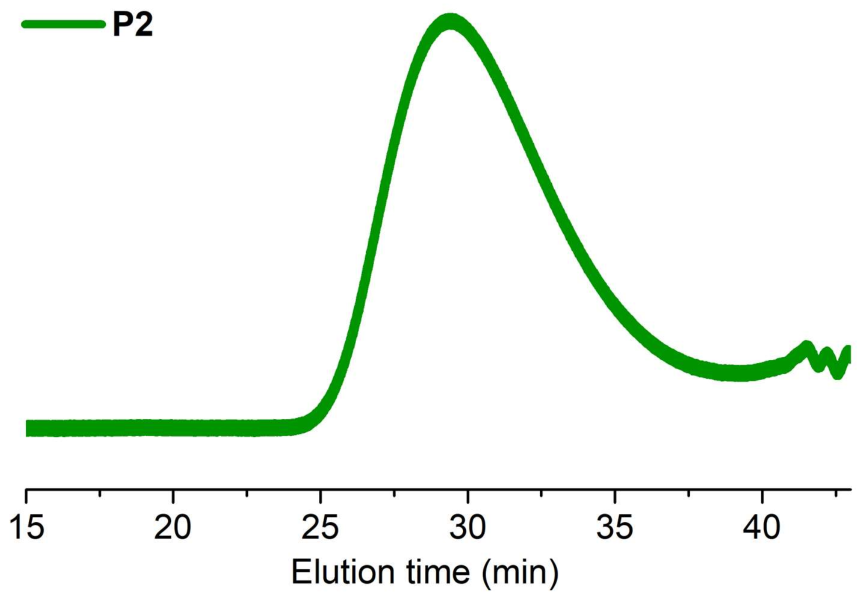

Figure S28. Size exclusion chromatography trace of P2.

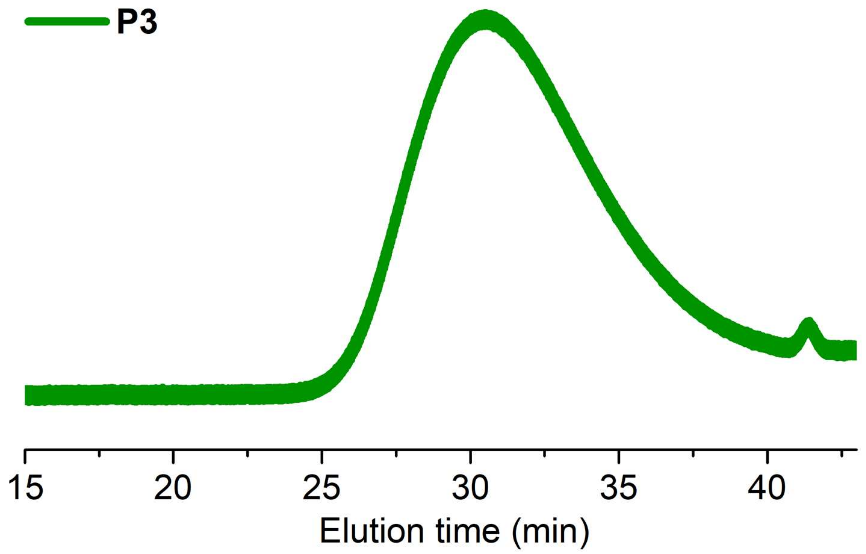

Figure S29. Size exclusion chromatography trace of P3.

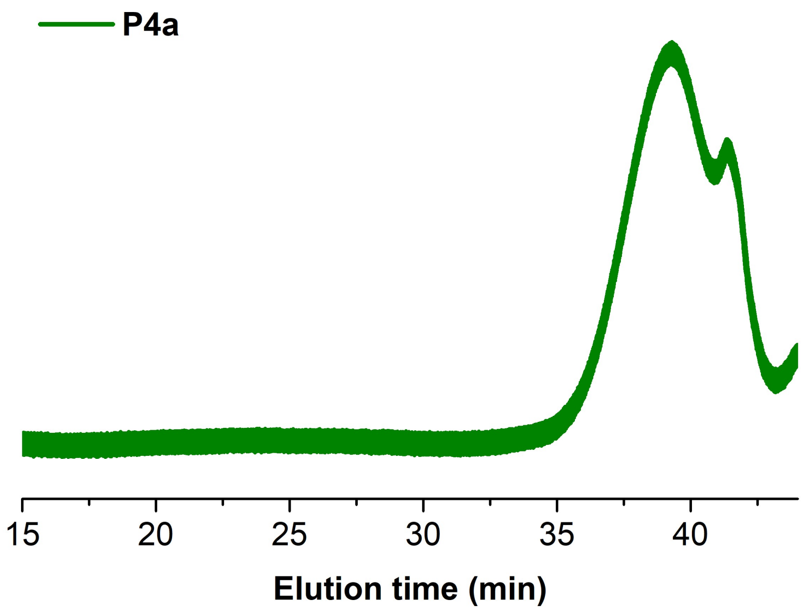

Figure S30. Size exclusion chromatography trace of P4a.

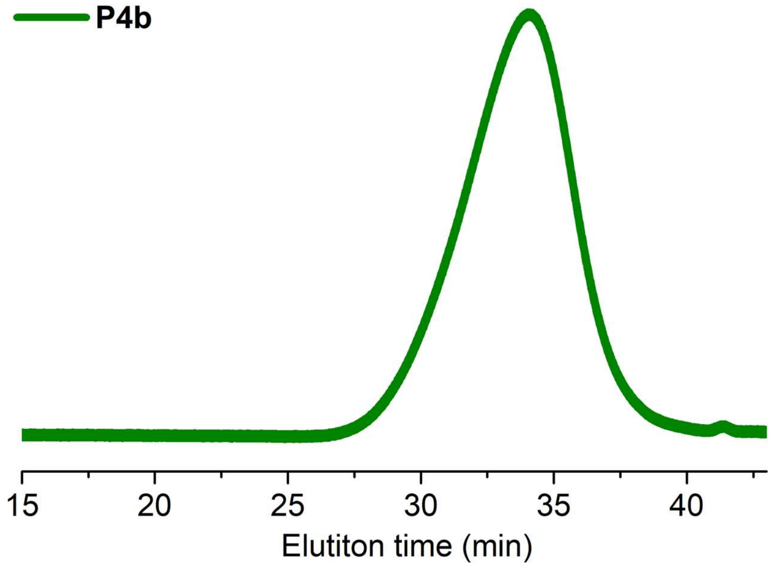

Figure S31. Size exclusion chromatography trace of P4b.

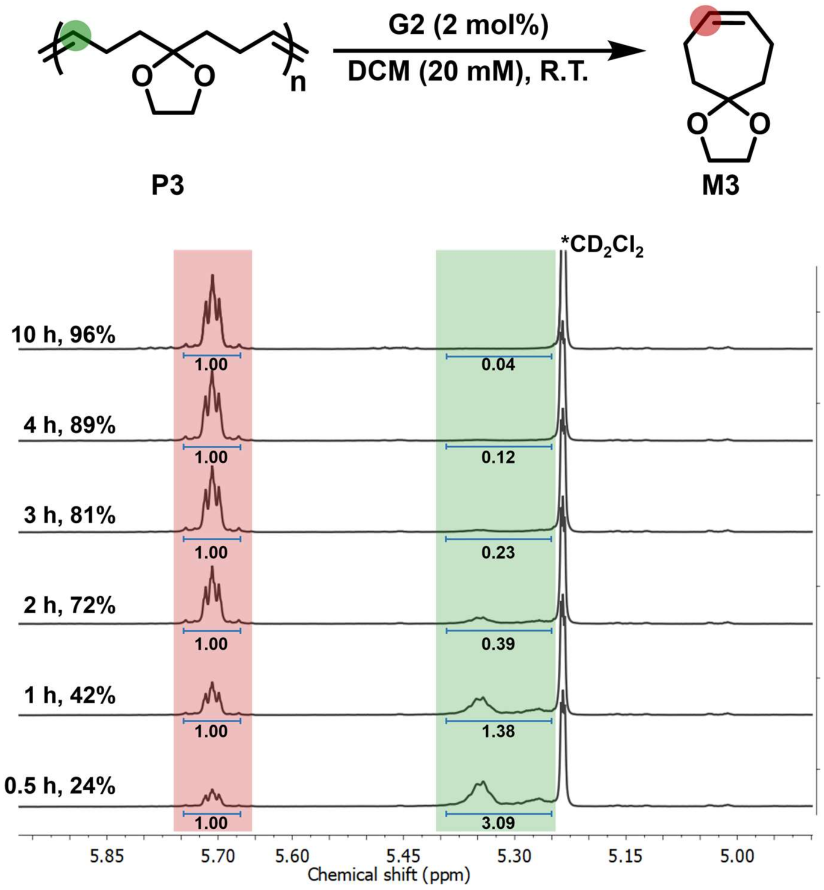

Figure S32. Depolymerization kinetics of P3 in CD_2Cl_2 at room temperature. Aliquots were collected at predetermined time points, and quenched with ethyl vinyl ether prior to ^{1}H NMR analysis.

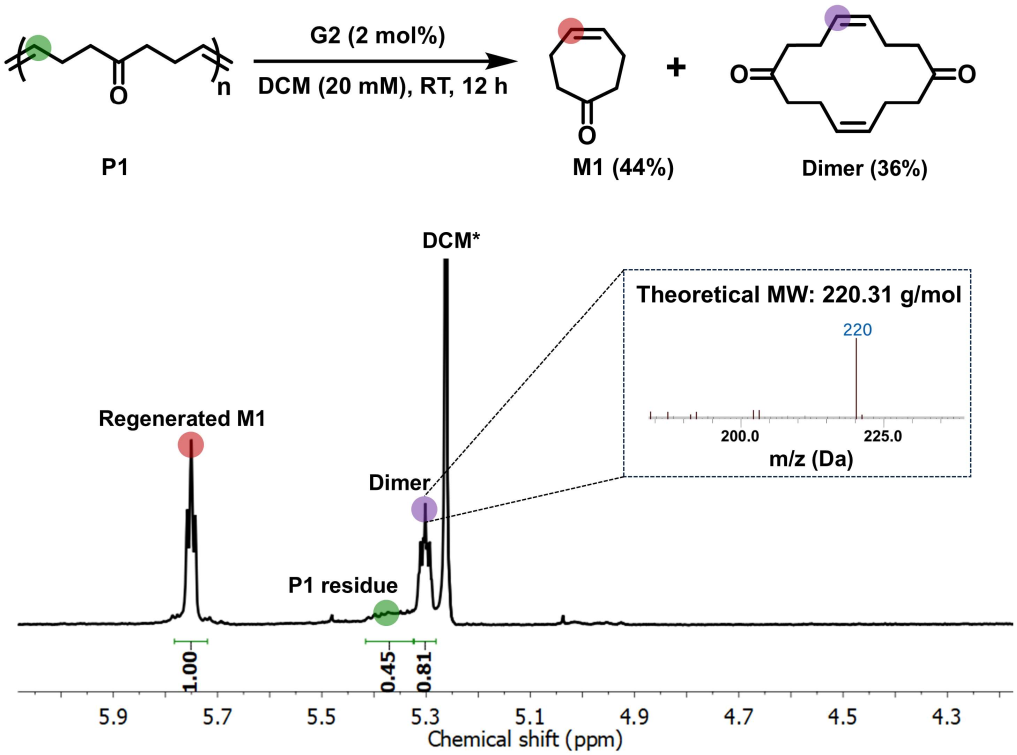

Figure S33. ^{1}H NMR analysis of depolymerized P1 in $CDCl_3$. The depolymerization was performed at room temperature for 12 h, leading to both M1 (44%), dimer (36%), and residual polymer/oligomer. GC-MS was used to confirm the structure of dimer.

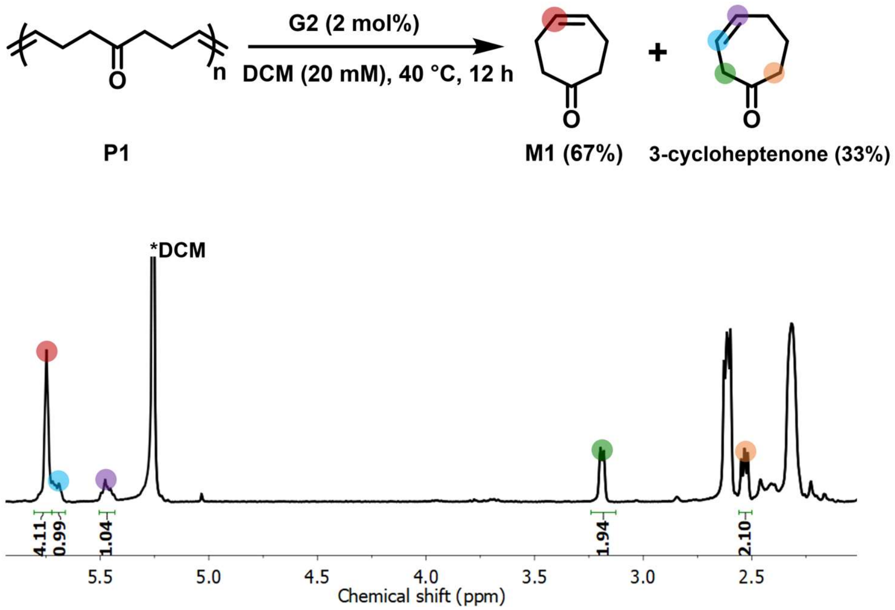

Figure S34. Partial ^{1}H NMR analysis of depolymerized P1 in $CDCl_3$. The depolymerization was performed in DCM at 40 °C for 12 h, yielding M1 (67%) and the thermally rearranged side product, 3-cycloheptenone (33%).

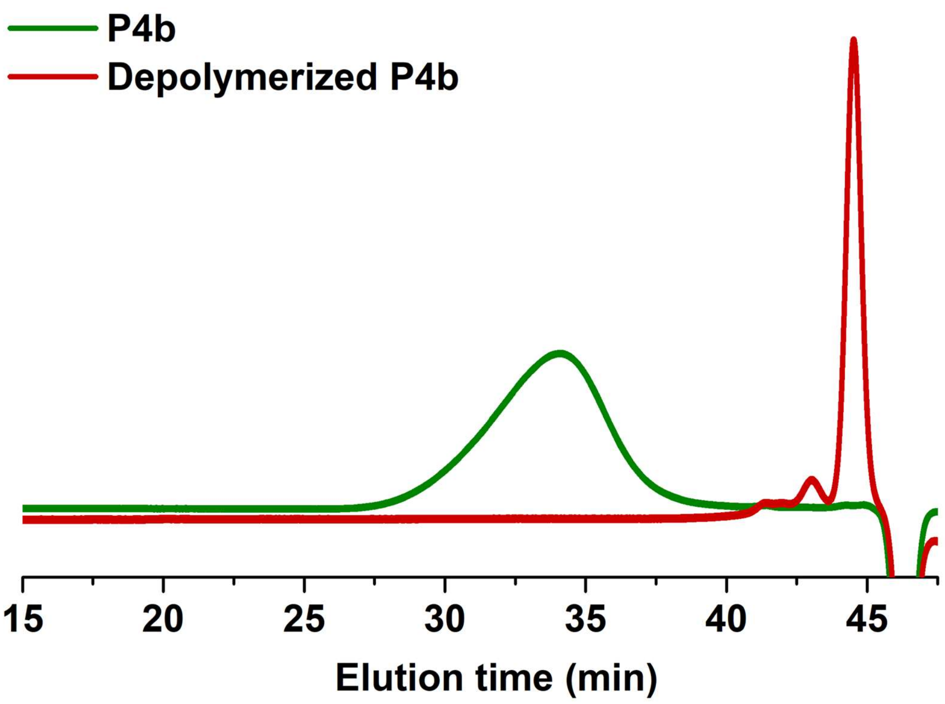

Figure S35. SEC traces of P4b and depolymerized product.

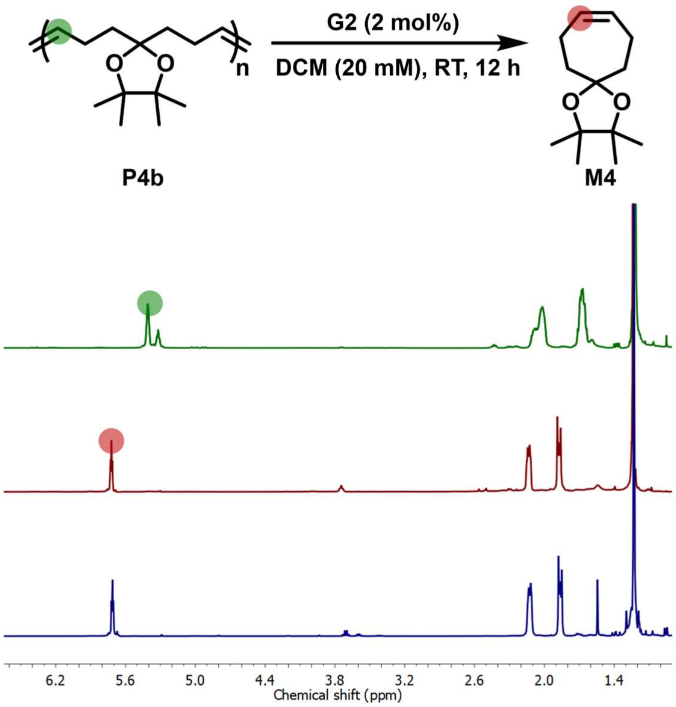

Figure S36. ^{1}H NMR spectra of P4b (top green), depolymerized product (middle red), and original M4 (bottom blue) in $CDCl_3$ at room temperature.

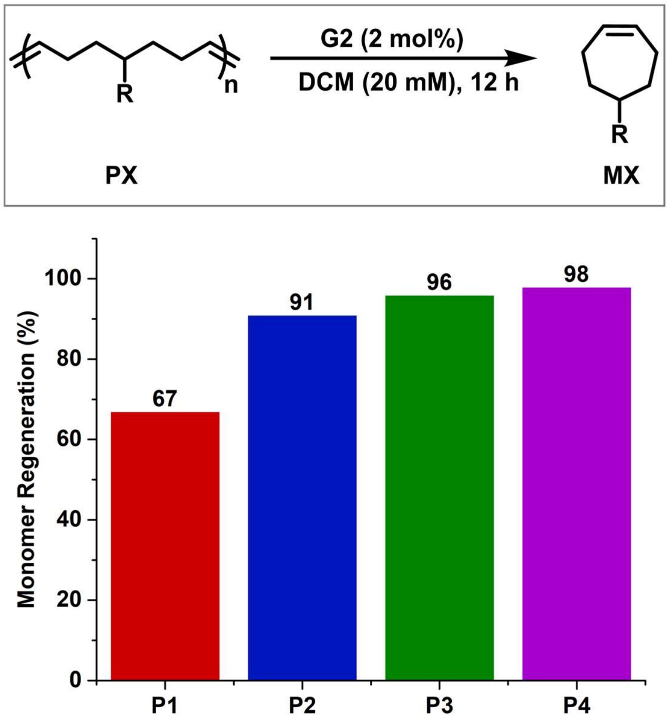

Figure S37. Depolymerization performance of P1, P2, P3, and P4b. The percentage of regenerated monomers was estimated by ^{1}H NMR. The depolymerization of P1 was conducted at 40 °C for 12 h. The depolymerizations of P2, P3, and P4b were conducted at room temperature for 12 h.

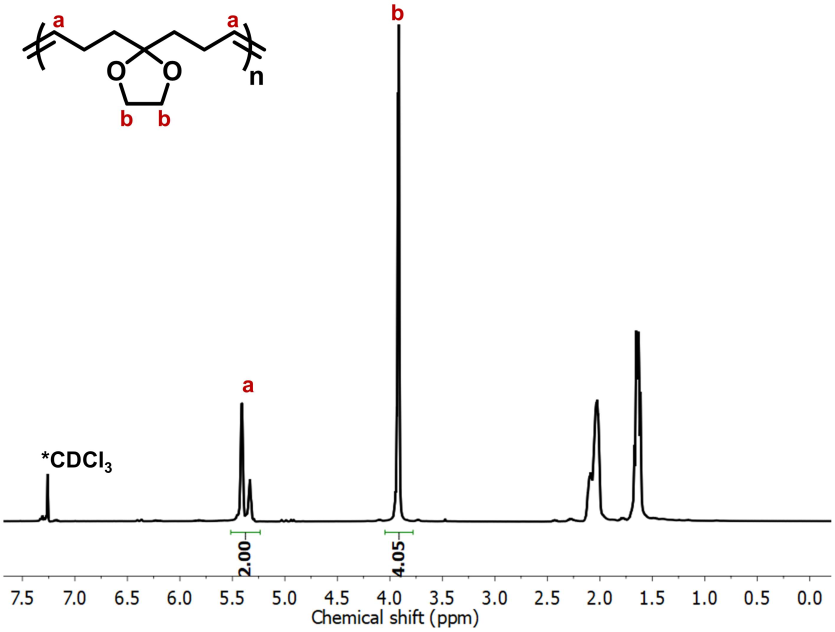

Figure S38. ^{1}H NMR spectrum of P3 obtained *via* post-polymerization modification of P1. The integration ratio of olefin (*a*) and methylene (*b*) signals in the cyclic acetal indicates a quantitative conversion of the ketones into the cyclic acetals alongside the polymer backbone.

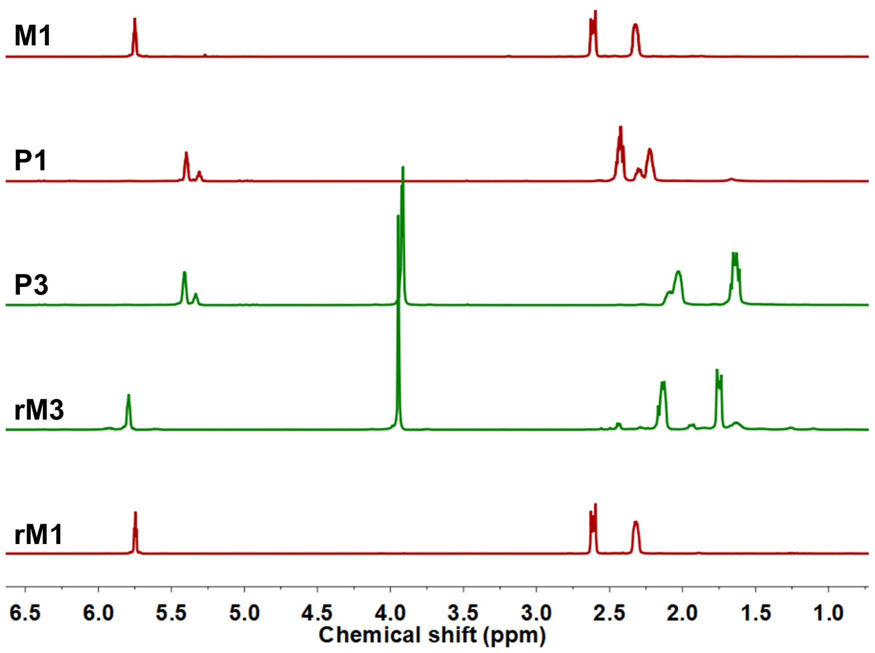

Figure S39. Full ^{1}H NMR spectra of M1, P1, P3 obtained *via* post-polymerization modification of P1, M3 from depolymerization, and regenerated M1(rM1) *via* hydrolysis of M3.

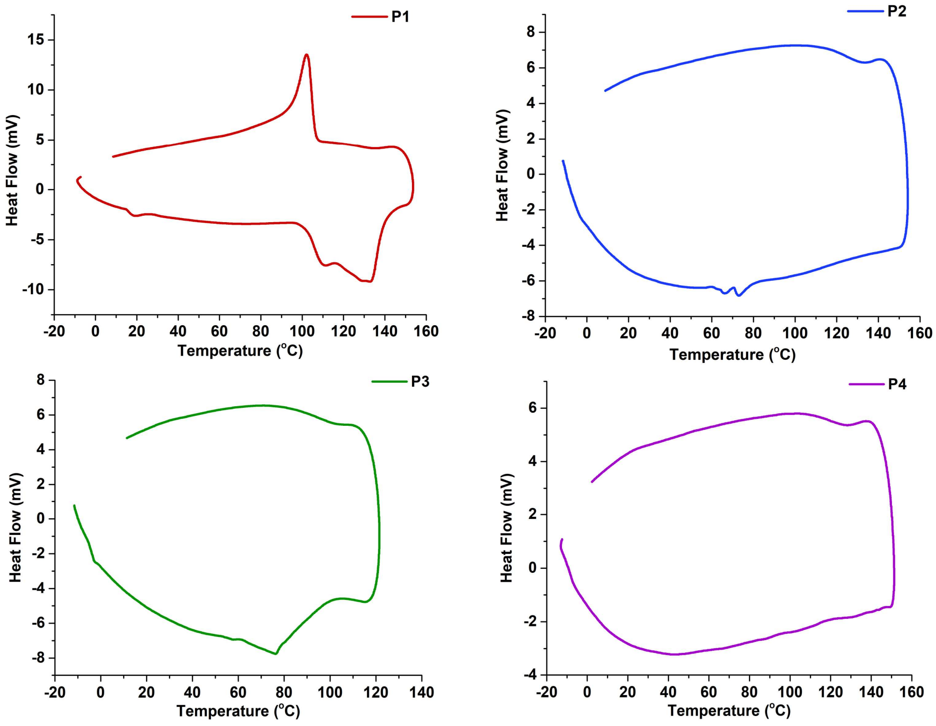

Figure S40. The first heating and first cooling scans of the DSC thermogram for P1-P4. Heating and cooling rates are 10 °C/min.

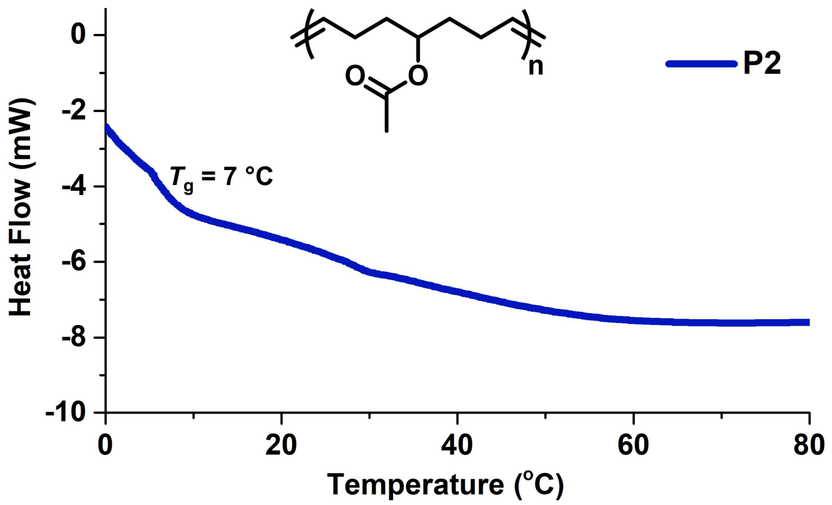

Figure S41. DSC thermogram of P2 (second heating scan). T_g value of P2 was determined by the midpoint of the sigmoidal change in the heat capacity.

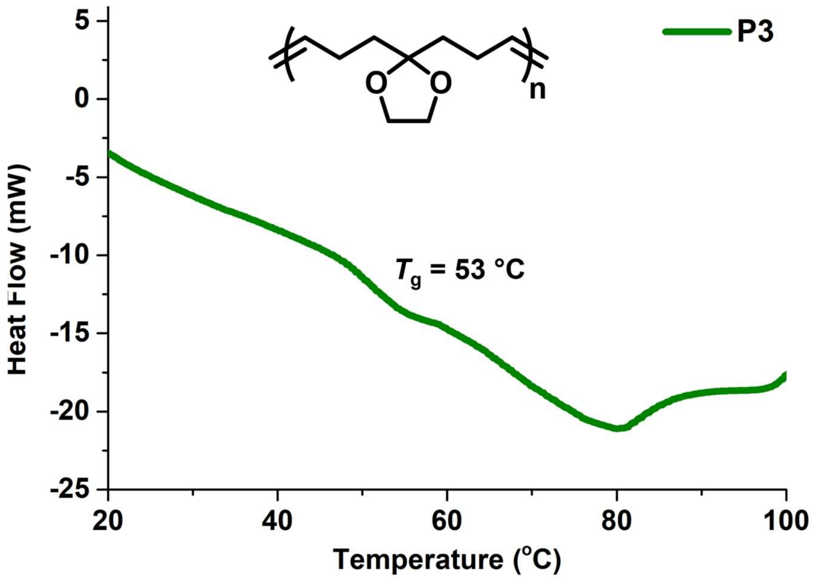

Figure S42. DSC thermogram of P3 (second heating scan). T_g value of P3 was determined by the midpoint of the sigmoidal change in the heat capacity.

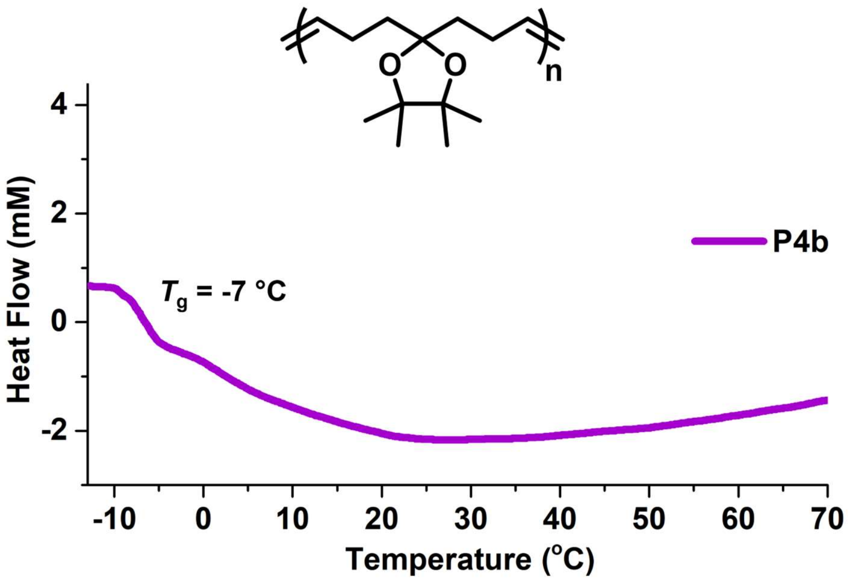

Figure S43. DSC thermogram of P4b (second heating scan). T_g value of P4b was determined by the midpoint of the sigmoidal change in the heat capacity.

5. Optimized Geometries of Monomers and Their Ring-Opened Structures from DFT Calculations

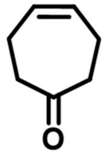

M1

C	1.827177	-0.008113	0.669130
C	1.827177	-0.008113	-0.669130
C	0.655872	0.335482	1.560341
C	0.655872	0.335482	-1.560341
C	-0.632300	-0.493050	1.306947
C	-0.632300	-0.493050	-1.306947
C	-1.322753	-0.126097	0.000000
O	-2.378990	0.481331	0.000000
H	2.752729	-0.275855	1.177214
H	2.752729	-0.275855	-1.177214
H	0.946095	0.186287	2.605855
H	0.403733	1.402469	1.467754
H	0.946095	0.186287	-2.605855
H	0.403733	1.402469	-1.467754
H	-0.372852	-1.559500	1.305594
H	-1.349976	-0.306355	2.111350
H	-0.372852	-1.559500	-1.305594
H	-1.349976	-0.306355	-2.111350

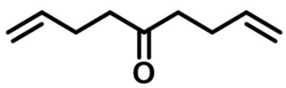

Ring-Opened M1

C	3.820607	-0.360786	-0.285060
C	2.560712	0.434077	-0.078192
C	1.296080	-0.415179	-0.274627
C	0.000231	0.378430	-0.134504
C	-1.297642	-0.411063	-0.278864
C	-2.560463	0.437128	-0.067491
C	-3.821439	-0.352979	-0.285832
C	4.764989	-0.558138	0.635473
C	-4.764802	-0.565255	0.632435
O	0.001914	1.576943	0.077036
H	3.946411	-0.805309	-1.274745
H	2.523486	1.275511	-0.782948
H	2.550673	0.876385	0.924166
H	1.296501	-0.891536	-1.267275
H	1.275616	-1.249207	0.441378
H	-1.302468	-0.874501	-1.277661
H	-1.275371	-1.254282	0.426078
H	-2.522984	1.289556	-0.758864
H	-2.549206	0.863300	0.941833
H	-3.948692	-0.780999	-1.282607
H	4.684839	-0.135014	1.635096
H	5.657090	-1.142475	0.426087
H	-5.657471	-1.145471	0.414241
H	-4.683371	-0.158907	1.638896

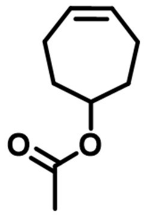

M2

C	2.947245	0.812046	0.265575
C	1.717577	1.624062	-0.074988
C	3.104410	-0.500649	0.063110
C	2.083866	-1.435183	-0.547345
C	0.392561	1.108819	0.522620
C	0.698280	-1.439360	0.130676
C	-0.129632	-0.179996	-0.116936
O	-1.446737	-0.473676	0.438919
C	-2.518536	0.091864	-0.162147
O	-2.453407	0.836479	-1.116762
C	-3.798309	-0.342596	0.516399
H	3.777031	1.361156	0.709706
H	1.865585	2.652808	0.271249
H	1.604997	1.694338	-1.168310
H	4.053999	-0.949719	0.352307
H	2.477291	-2.457281	-0.516417
H	1.954270	-1.200493	-1.615843
H	0.505019	0.956410	1.603365
H	-0.373862	1.877960	0.375782
H	0.814061	-1.593818	1.210656
H	0.121236	-2.289241	-0.254054
H	-0.258306	-0.019501	-1.192959
H	-4.647805	0.144834	0.036733
H	-3.907610	-1.430212	0.448979
H	-3.769532	-0.083708	1.579765

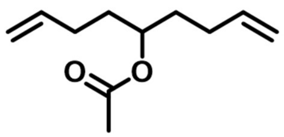

Ring-Opened M2

C	-4.686073	-1.675519	0.483083
C	-3.780240	-1.165110	-0.351703
C	-2.560522	-0.386856	0.061053
C	-1.253512	-1.056278	-0.401859
C	0.013555	-0.316955	0.033316
C	1.293801	-1.038353	-0.392204
C	2.585716	-0.360916	0.101086
C	3.822970	-1.098735	-0.332764
C	4.730754	-1.628667	0.487615
O	0.014142	1.000314	-0.591565
C	-0.051518	2.095456	0.206476
O	-0.125596	2.064072	1.414945
C	-0.013021	3.355209	-0.628689
H	-5.547503	-2.232580	0.124070
H	-4.603801	-1.550273	1.561148
H	-3.904938	-1.317141	-1.425911
H	-2.609200	0.622008	-0.372881
H	-2.547337	-0.260519	1.150982
H	-1.252523	-1.156842	-1.496088
H	-1.205837	-2.073754	0.007117
H	0.005553	-0.163023	1.116385
H	1.309876	-1.120249	-1.487810
H	1.252202	-2.062996	-0.000738
H	2.615463	0.663926	-0.295576
H	2.565927	-0.276133	1.195199
H	3.959448	-1.201911	-1.411234
H	5.605034	-2.154304	0.112696
H	4.637045	-1.552330	1.569342
H	0.934168	3.412242	-1.175732
H	-0.116194	4.224953	0.020957
H	-0.817204	3.344184	-1.371425

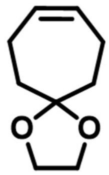

M3

C	2.694774	0.678078	-0.072478
C	1.494144	1.524690	-0.429380
C	0.270485	1.329421	0.489582
C	2.698269	-0.656346	0.016927
C	1.506011	-1.551663	-0.232639
C	0.272297	-1.267218	0.647298
C	-0.496801	0.014876	0.301331
O	-0.971452	-0.036413	-1.054799
C	-2.362633	-0.336969	-1.021829
C	-2.796451	0.275083	0.306000
O	-1.666539	0.035640	1.136827
H	3.623942	1.210420	0.128680
H	1.779374	2.582254	-0.393874
H	1.181051	1.328842	-1.464659
H	0.575528	1.398258	1.540183
H	-0.447523	2.137326	0.303848
H	3.630948	-1.151667	0.284864
H	1.802349	-2.593975	-0.070130
H	1.193597	-1.484322	-1.284383
H	0.559283	-1.224031	1.704744
H	-0.441353	-2.093388	0.543017
H	-2.833856	0.113929	-1.900171
H	-2.536787	-1.423379	-1.038944
H	-2.995554	1.352403	0.201832
H	-3.667637	-0.206203	0.759895

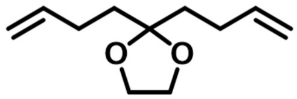

Ring-Opened M3

C	3.123104	-1.734330	-0.461049
C	2.326136	-0.482354	-0.213655
C	0.837157	-0.780958	0.032141
C	-0.036344	0.472617	0.165687
C	-1.489000	0.166189	0.571040
C	-2.267719	-0.708791	-0.429252
C	-3.726371	-0.813099	-0.074945
C	4.117106	-2.179369	0.308527
C	-4.372539	-1.944746	0.207452
O	0.008076	1.188039	-1.072270
O	0.506015	1.359325	1.161928
C	0.053062	2.575341	-0.756104
C	0.865020	2.589525	0.538067
H	2.837628	-2.313138	-1.342151
H	2.402234	0.180987	-1.085831
H	2.735646	0.058372	0.646768
H	0.441185	-1.386522	-0.792121
H	0.726120	-1.375858	0.946828
H	-1.479190	-0.304400	1.561278
H	-2.001885	1.128886	0.691517
H	-1.833579	-1.714452	-0.480622
H	-2.158767	-0.261786	-1.427651
H	-4.274129	0.131114	-0.043377
H	4.434393	-1.639260	1.198519
H	4.653569	-3.096809	0.080785
H	-5.429429	-1.953989	0.460552
H	-3.868432	-2.909328	0.191803
H	-0.957403	2.982916	-0.604456
H	0.528069	3.101092	-1.588689
H	0.609231	3.415388	1.209795
H	1.944339	2.617725	0.332339

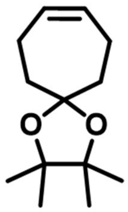

M4

C	3.602285	0.648799	0.073917
C	2.387465	1.543526	0.171237
C	1.240464	1.008425	1.050302
C	3.594667	-0.630571	-0.315177
C	2.372216	-1.415455	-0.732433
C	1.220706	-1.451524	0.295064
C	0.421283	-0.144311	0.439978
O	-0.110497	0.207081	-0.840333
C	-1.521788	0.487257	-0.730206
C	-1.919462	-0.386195	0.509730
O	-0.708754	-0.354070	1.293063
H	4.558224	1.098255	0.341356
H	2.694812	2.515684	0.573143
H	1.982088	1.742013	-0.831214
H	1.630626	0.672798	2.018765
H	0.542542	1.825292	1.259193
H	4.545008	-1.162842	-0.345978
H	2.670165	-2.450064	-0.938223
H	1.971453	-1.022185	-1.677275
H	1.606679	-1.725328	1.283977
H	0.515539	-2.232889	-0.002892
C	-1.732424	1.997275	-0.521753
H	-1.351898	2.340571	0.442964
H	-2.792957	2.266799	-0.582945
H	-1.197984	2.537638	-1.309502
C	-2.166021	0.074163	-2.055929
H	-1.806180	0.725073	-2.859540
H	-3.256477	0.172529	-2.006520
H	-1.917816	-0.955317	-2.321597
C	-3.035598	0.190856	1.382948
H	-2.777334	1.179459	1.767664
H	-3.210059	-0.468907	2.239179
H	-3.971810	0.264621	0.818016
C	-2.265769	-1.841285	0.148526
H	-1.534230	-2.282427	-0.532119
H	-3.253221	-1.912716	-0.321591
H	-2.281309	-2.437907	1.066081

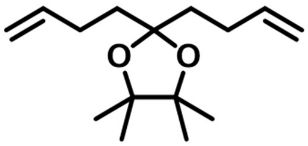

Ring-Opened M4

C	-0.585322	-1.145598	-0.911017
C	0.000000	0.000000	-0.063198
C	0.585322	1.145598	-0.911017
O	1.041493	-0.470049	0.794440
O	-1.041493	0.470049	0.794440
C	0.575736	-0.533538	2.158621
C	-0.575736	0.533538	2.158621
C	0.437721	-1.854290	-1.817885
C	-0.437721	1.854290	-1.817885
C	-0.134432	-3.088630	-2.460715
C	0.134432	3.088630	-2.460715
C	-0.253629	-3.292114	-3.773152
C	0.253629	3.292114	-3.773152
C	0.099747	-1.962812	2.473029
C	1.773411	-0.200179	3.052064
C	-1.773411	0.200179	3.052064
C	-0.099747	1.962812	2.473029
H	-1.027249	-1.872211	-0.221256
H	-1.414168	-0.753443	-1.511862
H	1.414168	0.753443	-1.511862
H	1.027249	1.872211	-0.221256
H	0.798934	-1.173490	-2.598178
H	1.306106	-2.126955	-1.201825
H	-1.306106	2.126955	-1.201825
H	-0.798934	1.173490	-2.598178
H	-0.482850	-3.861753	-1.772533
H	0.482850	3.861753	-1.772533
H	-0.680539	-4.207146	-4.175241
H	0.076374	-2.551008	-4.498981
H	0.680539	4.207146	-4.175241
H	-0.076374	2.551008	-4.498981
H	0.909741	-2.662580	2.243107
H	-0.773463	-2.250548	1.882806
H	-0.155874	-2.074337	3.532892
H	2.220122	0.758931	2.783172
H	1.479318	-0.170617	4.107558
H	2.541599	-0.972075	2.938745
H	-1.479318	0.170617	4.107558
H	-2.541599	0.972075	2.938745
H	-2.220122	-0.758931	2.783172
H	0.155874	2.074337	3.532892
H	-0.909741	2.662580	2.243107
H	0.773463	2.250548	1.882806

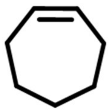

Cycloheptene

C	-0.150508	-1.545907	0.668801
C	-0.150508	-1.545907	-0.668801
C	0.326069	-0.428755	1.571838
C	0.326069	-0.428755	-1.571838
C	-0.286297	0.958992	1.301452
C	-0.286297	0.958992	-1.301452
C	0.170127	1.635561	-0.000000
H	-0.513309	-2.442248	1.171731
H	-0.513309	-2.442248	-1.171731
H	0.112597	-0.704978	2.611598
H	1.423498	-0.345471	1.507706
H	0.112597	-0.704978	-2.611598
H	1.423498	-0.345471	-1.507706
H	-1.381822	0.874747	1.305697
H	-0.021626	1.616640	2.140796
H	-1.381822	0.874747	-1.305697
H	-0.021626	1.616640	-2.140796
H	-0.199663	2.670235	-0.000000
H	1.269060	1.707065	-0.000000

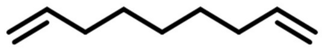

Ring-Opened Cycloheptene

C	-4.828591	0.432573	-0.283237
C	-3.798691	-0.414510	-0.273687
C	-2.551573	-0.256514	0.552833
C	-1.275965	-0.124626	-0.301466
C	0.000000	-0.000000	0.540355
C	1.275965	0.124626	-0.301466
C	2.551573	0.256514	0.552833
C	3.798691	0.414510	-0.273687
C	4.828591	-0.432573	-0.283237
H	-5.701550	0.269761	-0.910000
H	-4.839068	1.323841	0.341529
H	-3.831824	-1.294413	-0.919564
H	-2.650934	0.620782	1.206234
H	-2.438177	-1.128893	1.215296
H	-1.374245	0.750921	-0.957872
H	-1.191385	-0.997075	-0.965827
H	-0.085702	0.874535	1.202475
H	0.085702	-0.874535	1.202475
H	1.191385	0.997075	-0.965827
H	1.374245	-0.750921	-0.957872
H	2.438177	1.128893	1.215296
H	2.650934	-0.620782	1.206234
H	3.831824	1.294413	-0.919564
H	5.701550	-0.269761	-0.910000
H	4.839068	-1.323841	0.341529

6. References

[1] K. J. Arrington, C. B. Murray, E. C. Smith, H. Marand, J. B. Matson, *Macromolecules* **2016**, *49*, 3655-3662.
